# Bimetallic coupling in dual oxidant centres for the efficient removal of pharmaceuticals from wastewater: insights into the reaction mechanism

**DOI:** 10.1039/d6ra05958a

**Published:** 2026-09-08

**Authors:** Lalruatkima Ralte, Melody Lalhruaitluangi, Vanhmingliana, Barsha Rabha, Diwakar Tiwari

**Affiliations:** a Department of Chemistry, School of Physical Sciences, Mizoram University Aizawl-796004 India diw_tiwari@yahoo.com +91-9862323015

## Abstract

The widespread occurrence of pharmaceutical contaminants such as carbamazepine (CBZ) and norfloxacin (NFC) in aquatic ecosystems poses significant ecological hazards due to their persistence and bioactivity. The current study synthesizes novel bimetallic coupling Ce/Pd/ZY for the efficient degradation of pharmaceuticals using the photo-Fenton-like activation of H_2_O_2_ and persulfate (PS) under visible light irradiation. The catalyst exhibits a cubical structure with high crystallinity with a surface area of 36.04 m^2^ g^−1^ and average particle size of 8.6 nm, while optical and electrochemical characterization confirmed successful incorporation of nano Ce and Pd, enhanced light absorption, reduced band-gap energy (*E*_g_: 2.07 eV), and efficient photocurrent response, promoting efficient redox cycling between Ce^3+/^Ce^4+^ and Pd^0^/Pd^2+^. The synergistic activation of H_2_O_2_ and PS generated reactive oxygen species, predominantly ˙OH and SO_4_˙^−^ radicals, enabling rapid degradation of CBZ and NFC at neutral pH, achieving 86% and 96%, respectively. The degradation kinetics followed a pseudo-first-order model, showing that radical-driven oxidation is the rate-determining step. Intermediate identification suggested a successive hydroxylation, ring opening, defluorination, and progressive mineralization of pharmaceuticals. Acute toxicity assessment showed a high EC_50_ for the catalyst and a significant reduction in ecotoxicological risk after treatment of the EPs, confirming the catalyst's safety and the effective detoxification of the transformation products. The results highlight the potential of the Ce/Pd/ZY catalyst as a robust and sustainable catalyst for the advanced treatment of pharmaceutical-contaminated wastewater.

## Abstract 2

A novel bimetallic Ce/Pd/ZY catalyst was utilized in the efficient degradation of CBZ and NFC under UV-A and Vis-light irradiation, demonstrating excellent catalytic stability and low toxicity.

## Graphical abstract

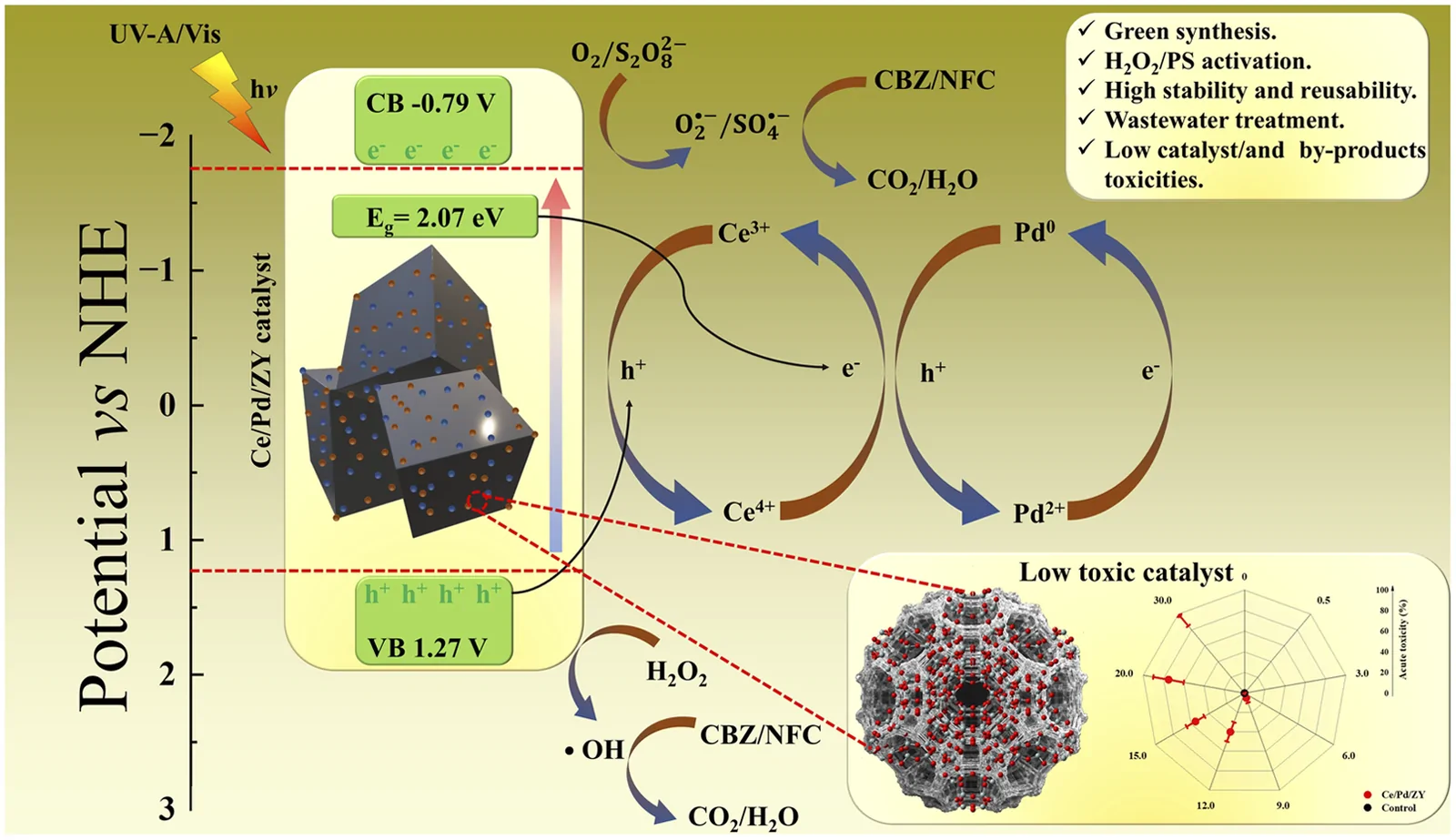

## 1. Introduction

Pharmaceutical compounds have raised severe environmental concerns due to their potential impact on the aquatic environment, even at low concentrations ranging from ng L^−1^ to µg L^−1^.^1–5^ Carbamazepine (CBZ) is a common anticonvulsant drug used globally, over 1010 tons per year, to treat epilepsy or other causes of seizures.^6^ The human body metabolises up to 72% of CBZ, while the remaining drug is excreted into the environment through the urine or faeces.^7,8^ The persistence of CBZ towards biodegradation, in conjunction with its robust lipophilicity and feeble biodegradation, enables it to withstand conventional wastewater treatment.^9,10^ Norfloxacin (NFC), a commonly prescribed fluoroquinolone antibiotic, is found to be recalcitrant in nature.^11^ A significant portion of NFC, approximately 75%, remains unmetabolized in the human body and is subsequently excreted, allowing it to enter and accumulate in aquatic environments more readily than many other antibiotics.^12^ The physicochemical stability, low biodegradability, and continuous discharge pose ecological and human health risks, necessitating efficient and sustainable treatment solutions.^13^

Advanced oxidation processes (AOPs) have received considerable attention for the degradation of the emerging pollutants (EPs) by the generation of highly reactive oxygen species (ROS) capable of mineralizing complex organic molecules. Among these, photo-Fenton-like processes, which activate hydrogen peroxide (H_2_O_2_) or persulfate (PS, S_2_O_8_^2−^) to generate high levels of ROS, are efficient for removing EPs under mild reaction conditions.^14,15^ However, the traditional Fenton process relies on H_2_O_2_ activation of Fe^2+^ to generate hydroxyl radicals (˙OH), but its applicability is often limited by a narrow pH range and the generation of excess iron sludge.^16^ However, several advancements are reported to overcome these limitations.^17,18^ Moreover, PS activation has emerged as an efficient alternative approach,^19^ relying on the sulfate radicals (SO_4_˙^−^) and ˙OH radicals, which exhibit strong oxidative capacity across a wider pH range and greater selectivity toward electron-rich contaminants, enabling tailored degradation pathways for several emerging water contaminants.^20^ Simultaneous use of H_2_O_2_ and persulfate provides a robust dual framework for degrading recalcitrant pharmaceuticals.^21^ These oxidative pathways, perhaps, break down aromatic rings, heterocycles, and halogenated groups present in CBZ and NFC, leading to significant reductions in toxicity and improved mineralisation efficiency.^22^ Continued advances of Fenton-like catalytic systems, including heterogeneous catalysts, metal–organic framework-doped carbons, metal oxides, bismuth oxyhalides and light-assisted Fenton-like processes, further enhance the selectivity and sustainability of these processes.^23–27^

Transition metals, such as copper, cobalt, manganese, nickel, chromium, and vanadium, have been explored as catalysts in Fenton-like processes; however, their potential toxicity poses severe environmental and health concerns.^28^ Copper and cobalt are among the most active alternatives for photocatalysts as well as peroxide or persulfate activation,^29,30^ but both are well known for their toxicity, even at low concentrations, which can cause oxidative stress, enzyme inhibition, and acute toxicity in organisms such as algae, daphnia, and fish.^31,32^ Nickel and chromium, particularly in their soluble or higher oxidation states, are associated with mutagenic and carcinogenic effects and persist in aquatic systems, posing long-term ecological risks.^33,34^ Vanadium and molybdenum, though effective redox catalysts, interfere with cellular metabolism and exhibit chronic toxicity toward aquatic biota upon accumulation.^35,36^ An essential trace ion, manganese, becomes neurotoxic and harmful to aquatic organisms at elevated concentrations.^37^ The leaching of these metals from homogeneous or poorly stabilized heterogeneous catalysts during Fenton-like reactions further exacerbates the risk of secondary contamination, undermining the environmental sustainability of the treatment process. Consequently, concerns regarding metal toxicity, leachability, and regulatory limits remain major challenges for the large-scale application of non-iron Fenton-like catalytic systems in water and wastewater treatment.

Standard ecotoxicological assays involving representative aquatic species, such as algae, daphnia, and fish, are necessary to determine potential acute and chronic effects associated with catalyst exposure. Such evaluations would provide critical information regarding the environmental compatibility of these materials and help bridge the existing gap between catalyst performance studies and real-world environmental risk assessment. Consequently, incorporating toxicity measurements into the design and evaluation of non-iron Fenton-like catalysts is crucial to developing safe, sustainable, and environmentally responsible wastewater treatment technologies.

Nevertheless, the current study focuses on developing a highly active catalyst supported on zeolite Y (ZY) using a green synthesis approach. Natural phytochemicals facilitate the *in situ* synthesis of the nano-ceria and Pd NPs within and across the pores of the zeolite framework. The heterojunction nanocomposite (Ce/Pd/ZY) is subsequently employed for the photo-Fenton-like degradation of EPs, notably CBZ and NFC, in an aquatic system utilizing visible and UV light across a broad pH range. Unlike conventional Fe-based and other transition-metal Fenton-like catalysts activating H_2_O_2_, Ce/Pd/ZY demonstrates enhanced catalytic activity through Ce-mediated redox cycling and Pd-assisted electron transfer within the zeolite-Y framework, with Pd promoting oxidant activation and enabling simultaneous activation of both H_2_O_2_ and persulfate under irradiation. Moreover, the catalyst's stability over successive operations further demonstrates its practical utility and cost-effectiveness.

A comprehensive investigation of the reaction mechanism and the robustness of the Ce/Pd/ZY catalyst provided greater insights into the process. In addition to evaluating the degradation performance of the Ce/Pd/ZY catalyst in real wastewater, the toxicity of the synthesized catalyst and the transformation intermediates formed during the degradation process were systematically investigated. This comprehensive approach addresses a critical research gap in Fenton-like catalysis, where catalyst toxicity and environmental risks associated with degradation by-products are often overlooked despite their importance for practical wastewater treatment applications.

## 2. Materials and methods

### 2.1. Chemicals

Cerium nitrate (Ce(NO_3_)_3_·6H_2_O) ≥ 99.9%, palladium nitrate (Pd(NO_3_)_2_), carbamazepine (CBZ), and norfloxacin (NFC) were procured from Sigma-Aldrich, USA. Propan-2-ol, sodium azide, hydrochloric acid (35.0%), and sodium hydroxide were obtained from Merck Co., Germany. Merck India Ltd supplied copper(ii) sulphate pentahydrate, nickel chloride, lead nitrate (99.0%), disodium hydrogen phosphate (99.0%), oxalic acid dihydrate, hydrogen peroxide (30%), sodium nitrate, and potassium persulfate. Glycine, manganese(ii) sulphate, and hydrogen peroxide (30%) were purchased from Fisher Scientific, India. The experimental solutions were prepared using purified water (conductivity: 4.0 µS cm^−1^; ionic concentration <1 µg L^−1^) obtained from a Millipore water purification system (Molsheim, France).

### 2.2. Extraction of phytochemicals

The papaya leaf (*C. papaya*) was first gathered from the Mizoram University campus, rinsed with distilled water, cut into small fragments, dried at ambient conditions, and then pulverized into a powder using a 100 BSS sieve. The finely ground powder (20 g) was heated with 200 mL purified water (PW) at 85 ± 3 °C for 15 min to obtain the leaf extract, which was then filtered through filter paper (11 µm). The phytochemicals used as reducing agents in the fabrication of the NPs are qualitatively analysed and presented in Table S1.

### 2.3. Preparation of Ce/ZY and Ce/Pd/ZY

**Scheme 1:**
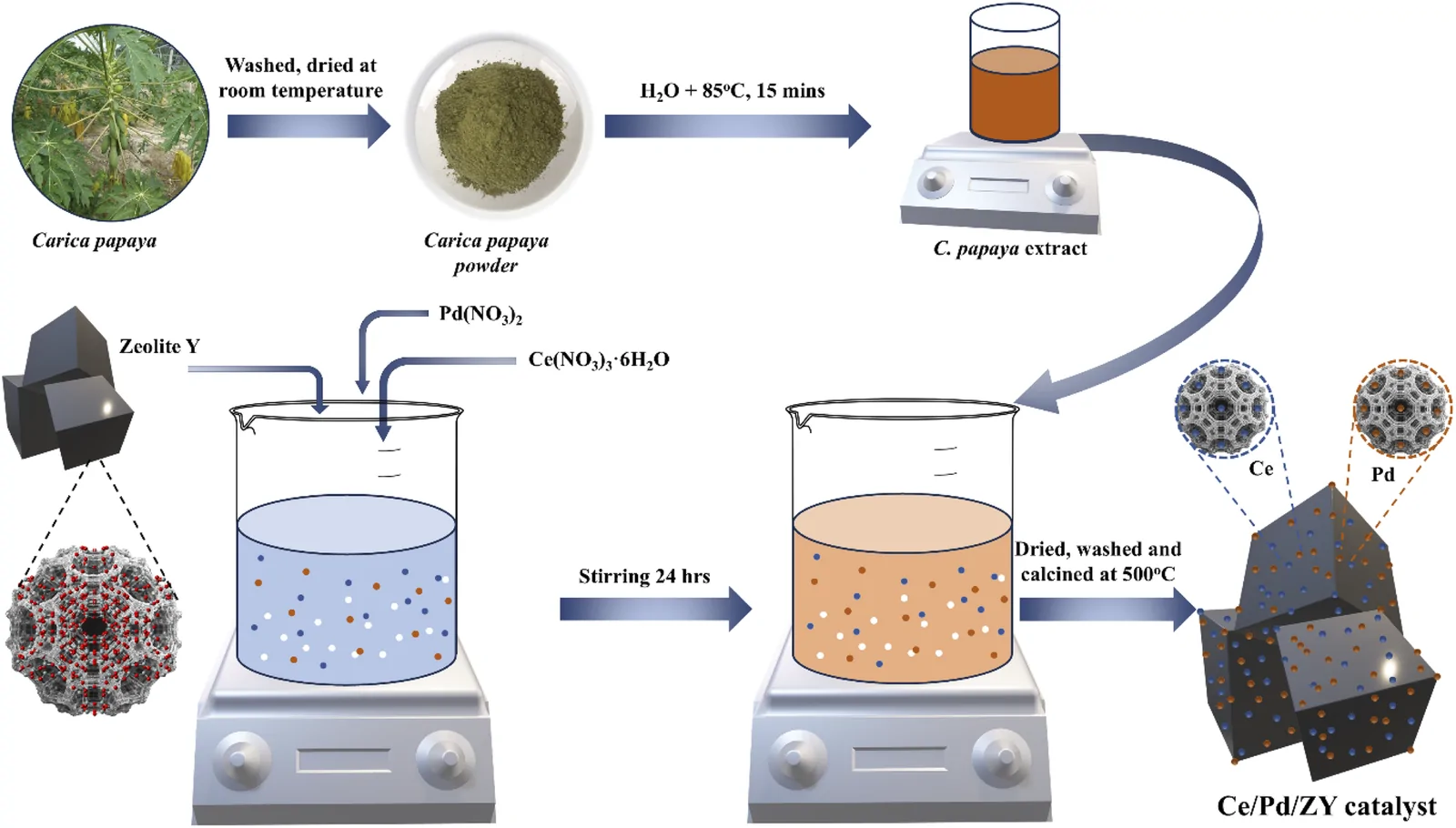
Synthesis process of Ce/Pd/ZY catalyst.

A 100 mL solution of purified water with optimize 0.1 M Ce(NO_3_)_3_·6H_2_O and 20 mL of 5 mM Pd(NO_3_)_2_ was stirred with 1 g of zeolite Y. After stirring the resultant mixture for 24 h, the freshly prepared and optimized amount of leaf extract (25 mL) was added dropwise, and the mixture was stirred for 30 min at 60 °C. Afterwards, the solid was rapidly obtained by centrifugation at 4000 rpm for 10 min. It was then washed five times with purified water, followed by ethanol. After a 12 h drying process in an oven, the solid was calcined for 3 h at 500 °C to ensure sufficient removal of residual phytochemicals and stabilization of the deposited metal nanoparticles, while preserving the crystalline structure of the zeolite Y framework and the catalyst was named Ce/Pd/ZY nanocomposite. The schematic diagram of the synthesis of Ce/Pd/ZY is depicted in Scheme 1. A similar process is used to synthesize Ce/ZY, without Pd, and the product was denoted as Ce/ZY.

### 2.4. Characterization of materials

Band-gap energy (*E*_g_) plays a crucial role in the photo-assisted Fenton-like process. Therefore, diffuse reflectance spectroscopy (DRS) provides better scattering phenomena in powder materials than UV-visible absorption spectroscopy, making it a more beneficial tool for assessing powdered samples.^38^ The *E*_g_ was calculated based on Kubelka–Munk's theory, which examines how light behaves as it passes through a light-scattering material.^39^ Using a DRS UV-visible spectrophotometer (EVOLUTION 220, Thermo Scientific, USA), the *E*_g_ was determined for the synthesized Ce/Pd/ZY, Ce/ZY, and zeolite Y. An electrochemical workstation (SP-200 Biologic Instrument, France) was used for the electrochemical impedance spectroscopy (EIS) and Mott–Schottky measurements. The studies used Ag/AgCl, platinum and glassy carbon electrodes as reference, counter, and working electrodes, respectively. A 0.004 M KCl solution was utilized as the electrolyte. The working electrode was modified by drop-casting a dimethylformamide solution containing the catalysts and dried in a hot air oven prior to the experiment.

The research utilized a thorough approach, employing a variety of advanced analytical techniques to give a thorough grasp of the catalysts. The surface morphology, particle shape, and particle size of the catalysts were assessed utilizing transmission electron microscopy (TEM; JEM 2100, JEOL, Japan) and field emission scanning electron microscopy (FE-SEM; JSM 7100F, JEOL, Japan). The composition and oxidation states of nanoceria and Pd nanoparticles were investigated using X-ray photoelectron spectroscopy (XPS; ESCALAB Xi+, Thermo Scientific Pvt. Ltd, UK). The X-ray diffraction (XRD; Philips X'pert MPD System, Netherlands) was utilized to examine the physical characteristics of catalysts. Additionally, the specific surface areas of Ce/Pd/ZY, Ce/ZY and zeolite Y were obtained using Brunauer–Emmett–Teller (BET; Micromeritics, ASAP 2010, USA).

### 2.5. Response surface methodology (RSM) for batch experiments

By analyzing and simulating the impacts of several factors, an RSM was utilized to enhance the degradation efficiency. Correlation-based RSM is employed to classify the contributing factors into low, medium, and high values and predict the degradation efficiencies.^40^ The statistical analysis of variance (ANOVA) was utilized for more accurate modelling along with the central composite design (CCD) to evaluate the accuracy and the effect of interaction factors on the degradation process, *viz.*, pH, catalyst dosage, H_2_O_2_ consumption and PS concentrations. To assess the model, 30 trials were designed using *F*-values, lack-of-fit tests, and *P*-values. The 3D surface response plots were generated to examine interactions among independent variables on optimum degradation efficiency, with a quadratic polynomial equation (eqn (S1) in the SI document) used to assess the influence and combined effects of each variable. Additionally, the coded variables are represented as *A*, *B*, *C* and *D* to simplify the analysis and interpretation of their individual and interactive effects.

### 2.6. Degradation of CBZ and NFC

To acquire the required concentrations for the studies, specific amounts of CBZ and NFC were dissolved in purified water (PW). The Ce/Pd/ZY catalyst was utilized in batch experiments to investigate the degradation of CBZ and NFC. Additionally, Ce/ZY and ZY were used to assess degradation performance compared with Ce/Pd/ZY.

Each experiment was carried out at room temperature in the dark, with a specific amount of catalyst added to a 100 mL EP solution, and the reaction mixture was continuously agitated for 30 min to reach adsorption saturation. The photo-Fenton-like reaction was initiated by adding a specific concentration of H_2_O_2_ and PS, followed by exposure to visible (Vis)/UV-A light (Vis: 50 W, Crompton, India; UV_*λ*max_: 360 nm, 9 W, Philips, South Korea). All the experiments were performed in triplicate. High-performance liquid chromatography (HPLC, Thermo Scientific) was used to determine the EPs removal efficiency at regular intervals. The CBZ and NFC degradation products were identified using liquid chromatography-mass spectrometry (LC-MS) (Agilent HPLC 1200 Series, Triple Quadrupole Mass Spectrometry System – API3200 AB SCIEX) using a typical C18 column (3.5 µm).

The degradation process was further investigated using real wastewater (RW) collected from a local wastewater treatment plant (SIPMIU), Mizoram, India. The quality of the real wastewater sample was further analyzed using a HI98914 multi-parameter (Hanna, USA). The water sample was used to produce varying amounts of CBZ and NFC to assess the nanocomposite catalyst's effectiveness in degrading the selected EPs during treatment. Additionally, the elimination of total organic carbon (TOC) was utilized for evaluating the mineralization efficiency of the EPs. Moreover, the catalyst's reusability was tested on both selected EPs in a continuous reactor operation after centrifugation, washing, and drying at 50 °C. The dried catalyst was then subjected to several subsequent trials.

### 2.7. Theoretical calculations

The structures of CBZ and NFC were optimized using the Gaussian 09 software package.^41^ The Becke hybrid functional (B3LYP) method is a widely acceptable method for analyzing the EPs.^42^ Thus, utilizing a triple zeta basis set for valence electrons, B3LYP/6-311+G(d,p) level theory, and a solvation model-based density (SMD) to mimic the solvent effects of water as the molecule, the molecular geometries and molecular orbital energies were determined.^43^ Additionally, the thermodynamic viability of the degradation process was further assessed by measuring the change in Gibbs free energy (Δ*G*). Utilizing the Multiwfn 3.8 software, the Fukui functions provided the reactive sites of CBZ and NFC. The molecular electronic structures of the reaction processes were visualized using the GaussView 6 and Multiwfn 3.8 programs utilizing the Fukui functions.

### 2.8. Toxicity tests

The toxicological study of the Ce/Pd/ZY was assessed utilizing *Daphnia magna* as the test organism. *Daphnia magna* was sourced from the National Institute of Environmental Research in the Republic of Korea. *Daphnia magna* was cultured in an M4 medium utilizing a 2 L glass beaker, following the standard OECD Guideline No. 211.^44^ The temperature was maintained at 20 ± 1 °C with a photoperiod of 16 h light and 8 h dark. The organisms were fed *Chlorella vulgaris* at a concentration of 5 × 10^5^ cells per mL every day, and the M4 medium was replenished twice weekly.

The acute toxicological study of the Ce/Pd/ZY was conducted following the OECD Guideline No. 202.^45^ Initially, various quantities of Ce/Pd/ZY solutions (0–25 mg L^−1^) were prepared for the test solution in an ISO medium (pH: 8; hardness: 250 mg L^−1^; DOC: 9.1 mg L^−1^).^46^ All the test solutions were filtered (Whatman filter (0.45 µm)). All studies, including the control group, were performed in four replicates, in which 20 individual neonate *Daphnia magna* were treated with 50 mL of the ISO medium for 48 h without feeding. The toxicity was evaluated by calculating the percentage of immobilization and mortality. The median effective concentration (EC_50_) and its 95% confidence interval (CI) were evaluated using the trimmed Spearman–Karber method.^47^

Furthermore, the toxicity of the selected EPs, *viz.*, CBZ and NFC, as well as the degradation by-products, was examined to assess the toxicity of compounds formed during degradation. The degradation by-products were identified by LC-MS, and their toxicity was assessed using ECOSAR V2.2 software. The software predicts the short-term (LC_50_/EC_50_) and long-term (ChV) toxicity of the by-products towards three organisms. The acute toxicity values were estimated in line with the European Union criteria, where the acute toxicity was classified based on LC_50_/EC_50_ values, where compounds with values exceeding 100 were considered not harmful, those within the range of 10–100 were categorized as harmful, values between 1 and 10 indicated toxic effects, and values below 1 were identified as highly toxic, whereas the chronic toxicity was assessed according to the Chinese hazardous compound evaluation guidelines, in which substances with ChV values greater than 10 were regarded as non-hazardous, those between 1 and 10 as harmful, values ranging from 0.1 to 1 as toxic, and values below 0.1 as highly toxic.^48,49^

## 3. Results

### 3.1. Characterization of Ce/Pd/ZY, Ce/ZY, and zeolite Y

**Fig. 1:**
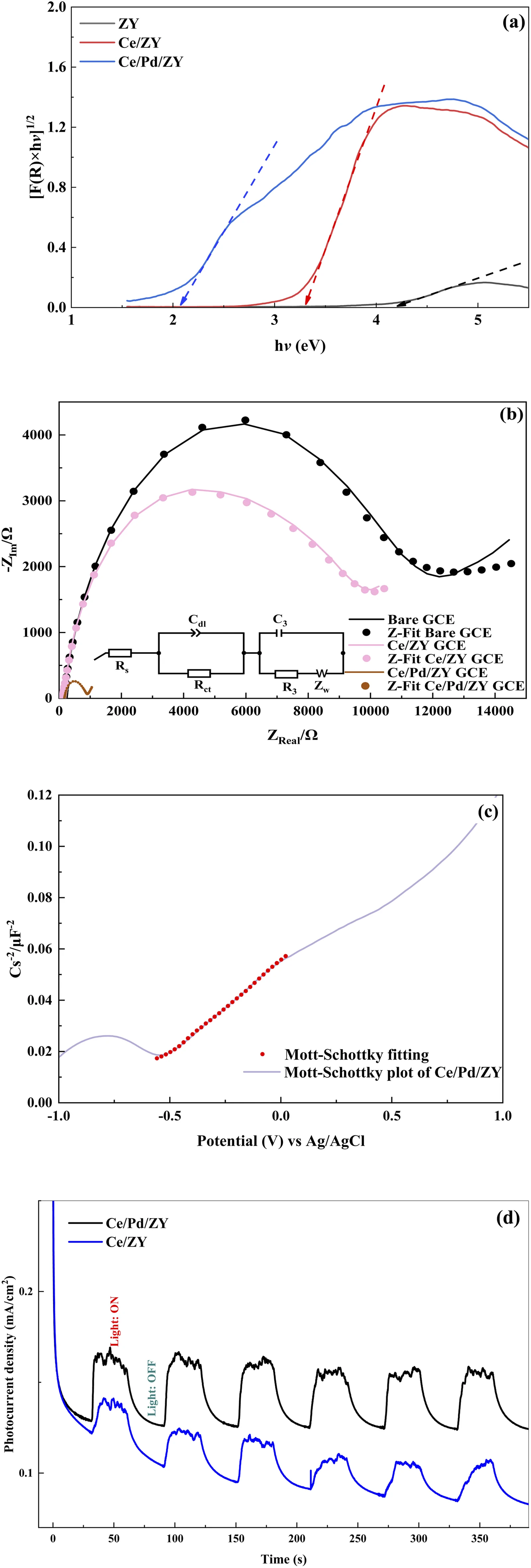
(a) Band gap energies of the catalysts; (b) EIS spectra of Ce/ZY and Ce/Pd/ZY solids [inset: the equivalent circuit diagram]; (c) Mott–Schottky plot of the Ce/Pd/ZY catalyst; and (d) photocurrent density measurement of the catalysts.

Diffuse reflectance spectroscopy (DRS) determines the *E*_g_ of ZY, Ce/ZY, and Ce/Pd/ZY catalysts. The *E*_g_ were estimated using the Tauc relation in conjunction with the Kubelka–Munk function to appropriately transform the DRS data.^50^ By plotting [*F*(*R*) × *hv*]^1/2^ as a function of photon energy (*hv*) according to the Tauc relation, the *E*_g_ of the catalysts were determined from the extrapolation of the linear region of the curve.^51^Fig. 1(a) compares the *E*_g_ of the ZY, Ce/ZY, and Ce/Pd/ZY catalysts obtained from the corresponding Tauc plots. The results revealed that the *E*_g_ of the Ce/Pd/ZY catalyst decreased to 2.07 eV, compared with 3.30 eV for Ce/ZY. The pristine zeolite Y exhibited an apparent optical absorption edge corresponding to approximately 4.21 eV. This absorption should not necessarily be interpreted as the conventional semiconductor band gap, as UV absorption in the zeolite Y has been associated with structural imperfections.^52^ These findings indicated that the incorporation of Pd NPs significantly narrowed the *E*_g_ of the Ce/Pd/ZY catalyst, which apparently facilitates the visible-light absorption capability, thereby promoting electron excitation through the generation of a localized electric field induced by the surface plasmon resonance (SPR) effect.^53,54^ Consequently, it promotes efficient degradation of EPs under visible-light irradiation.

Electrochemical impedance spectroscopy (EIS) and Mott–Schottky analyses were used to evaluate the interfacial electron-transfer resistance (*R*_ct_) and to determine the flat-band potential and carrier density of the catalysts. The EIS measurements were performed in a 0.004 M Fe^3+^/Fe^2+^ standard solution using a 0.1 M KCl background electrolyte solution.^46^ The resulting Nyquist plots are shown in Fig. 1(b). A simplified Randles equivalent circuit was employed to fit the EIS data, where *R*_s_ is the solution resistance, *R*_ct_ corresponds to the interfacial charge-transfer resistance, *C*_dl_ denotes the double-layer capacitance at the electrode–electrolyte interface and *Z*_w_ accounts for the diffusion-controlled mass transport [*Cf.* inset Fig. 1(b)].^55^ The EIS spectra exhibit semicircles in the high-frequency region, with the semicircle diameter corresponding to the *R*_ct_ at the solid–liquid interface.^46^ The bare glassy carbon electrode (GCE) was first measured for comparison. A substantial lowering of the *R*_ct_ value was observed for the Ce/Pd/ZY GCE (0.428 × 10^3^ Ω) in contrast to the Ce/ZY GCE (6.792 × 10^3^ Ω) and bare GCE (13.6 × 10^3^ Ω). These results show that incorporating Pd NPs significantly enhances charge transfer and charge separation while effectively suppressing charge recombination in the catalyst.^56^ The synergistic effect of nano ceria with Pd NPs also provides redox-active sites *via* a reversible Ce^3+/4+^ couple, along with oxygen vacancies.^57,58^ The comparative *R*_ct_ values are further shown in Table S2. The electrochemical properties of the Ce/Pd/ZY catalyst were further elucidated using Mott–Schottky analysis to identify the electronic band structure of the catalyst, as depicted in Fig. 1(c). The electrochemical Mott–Schottky analysis was performed over the potential range −1 to 1 V at 1000 Hz. A positive slope observed in the plot indicates that Ce/Pd/ZY behave as an n-type semiconductor, where e^−^ serve as the dominant charge carriers, while holes act as the minority carriers.^58^ For n-type semiconductors, the flat-band potential (*V*_fb_) is generally regarded as a reliable estimate of the conduction band (CB) potential.^59^ The Mott–Schottky analysis of the Ce/Pd/ZY showed a CB potential of −0.79 V, having a donor density of 1.97 × 10^27^ cm^−3^. Further, the valence band (VB) of the semiconductor was calculated from eqn (1). The calculated VB potential of Ce/Pd/ZY was found to be 1.27 V. Furthermore, the photochemical performance of the catalysts was evaluated in a sodium nitrate solution (0.1 M) at 0.5 V to examine the migration behaviour of photoinduced charge carriers and the recombination dynamics of electrons. The findings revealed that the photocurrent density of Ce/Pd/ZY is approximately twice more than that of the Ce/ZY catalyst [*Cf.*Fig. 1(d)], suggesting that the incorporation of Pd NPs significantly enhances the separation efficiency of photogenerated electron–hole pairs.^60^

1 *E*_VB_ = *E*_g_ + *E*_CB_

**Fig. 2:**
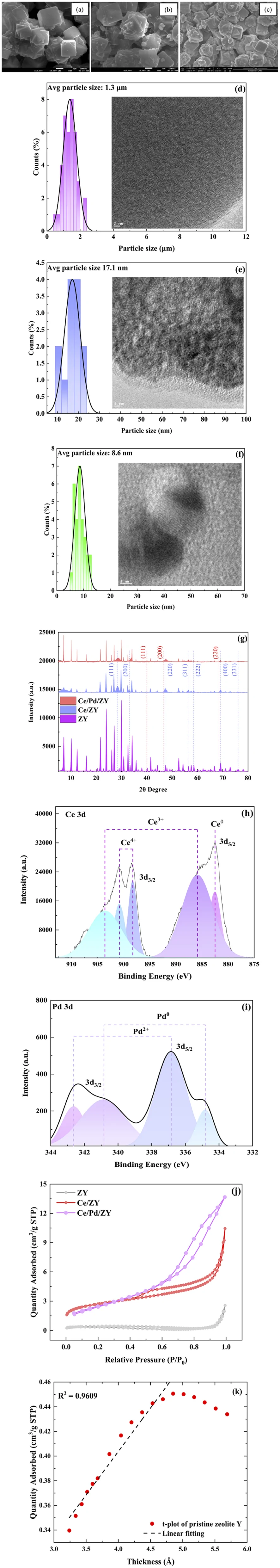
FE-SEM pictures of: (a) ZY; (b) Ce/ZY; and (c) Ce/Pd/ZY; particle size histogram of: (d) ZY; (e) Ce/ZY; and (f) Ce/Pd/ZY [inset: HR-TEM picture of the corresponding catalyst]; (g) XRD pattern of the ZY, Ce/ZY, and Ce/Pd/ZY catalysts; high resolution XPS of: (h) Ce 3d; (i) Pd 3d; (j) N_2_ isotherms of ZY, Ce/ZY, and Ce/Pd/ZY catalysts; and (k) *t*-plot of the pristine zeolite Y.

The field-emission scanning electron microscopy (FE-SEM) images of ZY, Ce/ZY, and Ce/Pd/ZY solids reveal the morphology of these solids and are shown in Fig. 2(a)–(c). The images demonstrate that ZY exhibits a highly crystalline structure with distinct cubic shapes, and that its surface features several micropores and sharp edges. In contrast, the Ce/ZY catalyst exhibits a more compact, irregular surface structure, attributed to the successful fabrication of nanoceria on the surface and within the pores of ZY. Moreover, the FE-SEM image further shows that the surface of ZY in the Ce/Pd/ZY catalyst is predominantly covered with nano ceria and Pd NPs [*Cf.*Fig. 2(c)], resulting in a more heterogeneous and disordered morphology. This heterogeneity and disordered morphology increase the surface roughness, which potentially increases the defect density and specific surface area of nano ceria, affecting its chemical properties.^61^ The zeolite Y, acting as a support, ensures the uniform distribution of small-sized ceria and Pd(NPs), limits agglomeration, and increases the number of active sites, thereby significantly improving the catalyst's performance.^62^ Similarly, the enhanced stability and superior catalytic performance of Pt-incorporated zeolite Y were reported elsewhere.^63^ Moreover, the EDX spectra are shown in the inset of the corresponding SEM images, and the quantitative elemental compositions are provided in Table 1, confirming the successful incorporation of both Ce and Pd into the zeolite framework, with 1.01 and 4.80 wt%, respectively, in the Ce/Pd/ZY catalyst.

**Table 1:** Quantitative EDX analysis of Ce/ZY and Ce/Pd/ZY catalysts

Element	Weight (%)	
	Ce/ZY	Ce/Pd/ZY
C	17.27	11.72
O	42.8	45.53
Na	4.88	5.57
Al	11.09	12.18
Si	11.62	13.14
K	5.4	5.95
Ca	0.05	0.1
Pd	—	1.01
Ce	6.89	4.8

The high-resolution transmission electron microscopy (HR-TEM) pictures of the zeolite Y, Ce/ZY, and Ce/Pd/ZY, along with the particle size histogram, are depicted in Fig. 2. The pristine ZY displays a crystalline Faujasite-type structure with an average particle size of 1.3 µm [*Cf.*Fig. 2(d)]. It is apparent that the microporous structure of ZY provides an ideal support for NPs to be distinctly and uniformly distributed without agglomeration, as shown in Fig. S1. Evidently, the HR-TEM images of Ce/ZY and Ce/Pd/ZY catalysts display a regular dispersion of nanoceria and Pd NPs across the surface and microporous framework of ZY [*Cf.*Fig. 2(e) and (f)]. The average metal nanoparticle size of the Ce/ZY and Ce/Pd/ZY is 17.1 nm [*Cf.*Fig. 2(e)] and 8.6 nm [*Cf.*Fig. 2(f)], respectively. The *d*-spacing values are measured with ImageJ software as depicted in Fig. S2, where the values of ZY, Ce/ZY, and Ce/Pd/ZY were 0.65, 0.29 and 0.22 nm, respectively. These values correspond to the (311) plane of the Faujasite structure of zeolite Y (ZY), while the (200) and the (111) planes of the face-centred cubic (fcc) structures of nano ceria and Pd NPs.^55,58^

The X-ray diffraction (XRD) results of the ZY, Ce/ZY, and Ce/Pd/ZY solids are shown in Fig. 2(g). The diffraction patterns of the zeolite Y follow the standard JCPDS no. 43-0168 for zeolite Y (FAU structure), with characteristic peaks observed at 2*θ* values of 7.2, 10.2, 12.4, 16.1, 20.3, 23.9, and 27.1, confirming the presence of the Faujasite framework.^58^ Furthermore, similar diffraction patterns are observed for Ce/ZY and Ce/Pd/ZY catalysts, indicating that the crystalline framework of zeolite Y remains structurally stable after metal incorporation. However, additional diffraction peaks are observed and are attributed to the formation of nanoceria and Pd NPs. The diffraction peaks at 2*θ* values of 28.35, 33.26, 47.81, 56.40, 58.60 and 69.20° are indexed to the (111), (200), (220), (311), (222), and (400) planes of the face-centred cubic structure of nanoceria (JCPDS No. 34-0394).^64^ In addition, the peaks at 40.05, 46.75, and 68.54° correspond to the (111), (200), and (220) planes of the face-centred cubic structure of Pd NPs (JCPDS No. 05-0681).^65^

The X-ray photoelectron spectroscopy (XPS) was utilized to examine the surface composition and oxidation states of the nanoceria and Pd NPs in the Ce/Pd/ZY catalyst. Fig. S3 shows the XPS characteristic peaks of Ce 3d, Pd 3d, C 1s, and O 1s, confirming the successful incorporation of nanoceria and Pd NPs. Moreover, Fig. 2(h) and (i) present the high-resolution XPS spectra of Ce 3d and Pd 3d in the Ce/Pd/ZY solid, respectively. The Ce 3d spectrum shows two distinct peaks of Ce 3d_5/2_ and Ce 3d_3/2_, along with their associated satellite peaks. The peaks at 885.7 eV and 903.0 eV correspond to Ce^3+^ species, while the peaks at 898.2 eV and 900.8 eV correspond to Ce^4+^, indicating the coexistence of Ce^3+/4+^ oxidation states on the catalyst surface.^66^ Moreover, the photoelectron peak at 883.0 eV indicates the presence of reduced cerium species in the Ce/Pd/ZY catalyst.^67^ Nevertheless, the deconvoluted XPS spectrum of Pd 3d exhibits two main peaks at binding energies of 335.2 eV and 340.3 eV, corresponding to Pd 3d_5/2_ and Pd 3d_3/2_, respectively.^68^ The components at 334.8 eV and 340.7 eV are assigned to metallic Pd^0^ species, while the peaks at 336.8 and 342.6 eV are attributed to Pd^2+^ species.^69^ The quantitative peak fitting parameters are presented in Table S15. The presence of Ce^0/3+/4+^ and Pd^0/2+^ is crucial to enhancing the Fenton-like catalytic performance of the Ce/Pd/ZY catalyst in activating both H_2_O_2_ and PS. The reversible redox cycling between nano ceria and Pd NPs facilitates continuous electron transfer reactions and enhances the efficient activation of H_2_O_2_ into highly reactive ˙OH radicals, which accelerate the decomposition of PS into active sulfate radicals. The synergistic interaction between the two metal NPs thus increases radical generation and sustains catalytic redox cycles, which ultimately improve degradation efficiency towards EPs.

The N_2_ adsorption isotherms of ZY, Ce/ZY, and Ce/Pd/ZY are shown in Fig. 2(j) after degassing at 200 °C for 4 h at 50 mTorr. The pristine ZY exhibits lower N_2_ uptake, indicating limited accessible porosity, consistent with its lower BET surface area of 1.61 m^2^ g^−1^ and small pore volume of 0.0041 cm^3^ g^−1^. In contrast, both Ce/ZY and Ce/Pd/ZY display typical Type IV isotherms with an H3 hysteresis loop observed at relative pressure above 0.4 *P*/*P*_0_, highlighting their mesoporous structures, with characteristic pores formed by interparticle voids arising from the aggregation of metal NPs on the zeolite surface. The BET surface area and pore volume were increased significantly from 9.79 m^2^ g^−1^ and 0.0164 cm^3^ g^−1^ for Ce/ZY to 36.04 m^2^ g^−1^ and 0.0819 cm^3^ g^−1^ for Ce/Pd/ZY, accompanied by a decrease in average pore size from 65.0 to 45.5 Å. In addition, the *t*-plot analysis revealed a micropore surface area of 0.7199 m^2^ g^−1^ and a micropore volume of 0.000279 cm^3^ g^−1^ of pristine zeolite Y. The analysis was performed using the Harkins–Jura thickness equation with a linear fitting range of 3.50–5.02 Å depicted in Fig. 2(k). The changes in surface area, pore volume, and pore size suggest that the synergistic incorporation of nano ceria and Pd NPs yields a hierarchical mesoporous structure with enhanced surface area and pore accessibility, thereby increasing catalytic activity. A comparative table of the BET surface area, pore volume, and pore size for ZY, Ce/ZY, and Ce/Pd/ZY is shown in Table S3.

### 3.2. RSM-CCD modelling and optimization of the photo-Fenton-like system in the elimination of CBZ and NFC

RSM-CCD was used to evaluate interactions among the independent factors (*A*: pH, *B*: catalyst dosage, *C*: H_2_O_2_ concentration, and *D*: PS concentration) for CBZ and NFC removal. Analysis of variance (ANOVA) was utilized to evaluate the statistical significance and adequacy of the quadratic regression models, as well as to assess the individual and interactive effects of the process variables on the removal of these two pollutants.^46^ Fisher's test yielded *F*-values of 106.91 for CBZ/UV-A and 218.59 for NFC/UV-A, 104.10 and 132.70 for CBZ/Vis and NFC/Vis, indicating that the selected regression models are highly significant and appropriate for describing the experimental data. Furthermore, the *p*-value of 0.0001 for the elimination of both EPs under UV-A and Vis illuminations is significantly lower than the 0.05 significance level, demonstrating the statistical significance of the models at a 95% confidence level and indicating a negligible margin of error. The contributions of each factor were also evaluated to determine their influence on the overall response. A strong correlation between the predicted and experimental degradation efficiencies of CBZ and NFC is observed, as reflected by high coefficients of determination (*R*^2^ = 0.9901 for CBZ/UV-A, 0.9951 for NFC/UV-A, 0.9898 for CBZ/Vis, and 0.9920 for NFC/Vis). Additionally, the adjusted *R*^2^ values of 0.9808, 0.9906, 0.9803, and 0.9845 for CBZ/UV-A, NFC/UV-A, CBZ/Vis, and NFC/Vis, respectively, further confirm the model's robustness after excluding insignificant terms. The precision levels of 26.08 for CBZ/UV-A, 37.43 for NFC/UV-A, 26.50 for CBZ/Vis, and 29.74 for NFC/Vis further demonstrate the model's reliability, as values greater than 4 indicate an adequate signal-to-noise ratio. The ANOVA analysis for the removal of CBZ under the different light irradiations is shown in Tables S4 and S5, respectively, while Tables S6 and S7 summarize the corresponding ANOVA analysis of NFC removal.

Tables S8 and S9 show the performance of the Ce/Pd/ZY catalyst for the removal of CBZ under the two light conditions, while Tables S10 and S11 report the analogous results for NFC. The experiments were designed over a wide pH range of 2–12, with catalyst dosage from 50–200 mg L^−1^, H_2_O_2_ (30%) concentration of 0.1–0.2 mL L^−1^ and PS concentration of 150–210 mg L^−1^. The relationships between the coded variables and the degradation efficiencies of CBZ/UV-A, NFC-UVA, CBZ/Vis, and NFC/Vis are described by eqn (2)–(5), respectively. The pH of the solution was identified as the primary factor affecting the degradation of both the EPs. The magnitude of deviation from the reference point indicates the relative significance of each factor. The influence of the factors *A*, *B*, *C* and *D* on the experimental degradation efficiencies of the EPs was further evaluated utilizing three-dimensional response surface plots.

2 *Y*_CBZ/UV-A_ = 78.8 − 6.1*A* + 2.9*B* − 1.1*C* − 0.1*D* − 1.2*AB* − 0.3*AC* + 0.1*AD* + 0.006*BC* − 0.5*BD* + 0.3*CD* − 53.4*A*^2^ + 3.0*B*^2^ + 5.4*C*^2^ − 6.5*D*^2^

3 *Y*_NFC/UV-A_ = 93.6 − 2.8*A* − 3.1*B* − 0.2*C* − −0.09*D* − 0.1*AB* + 0.5*AC* + 0.09*AD* − 0.02*BC* + 0.8*BD* − 0.3*CD* − 26.1*A*^2^ − 3.9*B*^2^ − 4.0*C*^2^ + 0.5*D*^2^

4 *Y*_CBZ/LED_ = 70.1 − 5.5 + 2.9*B* − 1.2*C* − 0.5*D* − 1.1*AB* + 0.07*AC* + 0.6*AD* + 0.3*BC* − 1.0*BD* + 0.1*CD* − 52.2*A*^2^ + 3.8*B*^2^ + 6.2*C*^2^ − 4.4*D*^2^

5 *Y*_NFC/LED_ = 83.0 − 2.9*A* − 2.6*B* − 0.5*C* − 0.1*D* − 0.3*AB* + 1.0*AC* − 0.08*AD* − 0.1*BC* + 1.2*BD* − 0.3*CD* − 25.6*A*^2^ − 3.8*B*^2^ − 4.5*C*^2^ + 1.1*D*^2^

**Fig. 3:**
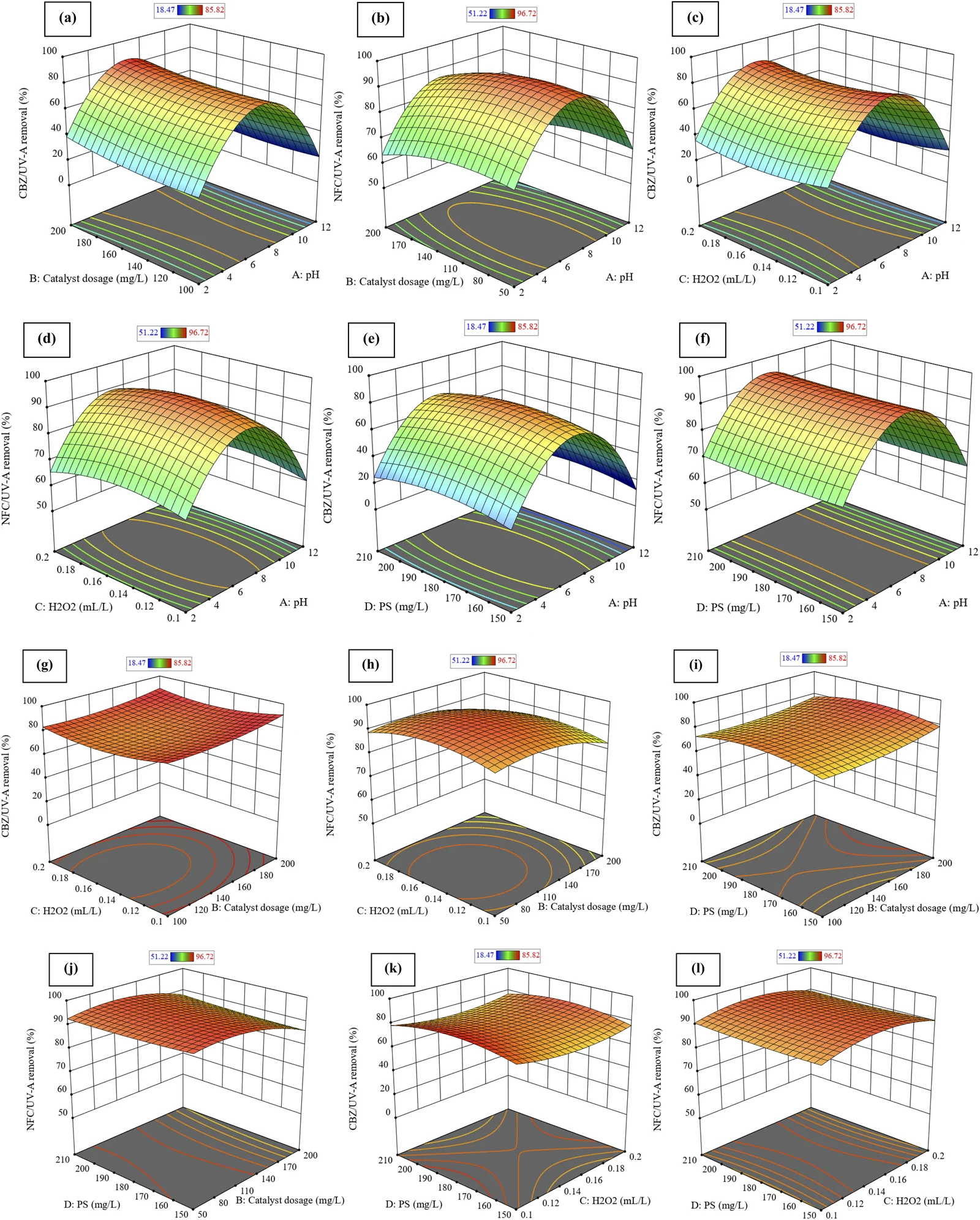
Degradation efficiency of CBZ and NFC under UV-A utilizing 3D surface plot across two factors: (a) pH *vs.* catalyst dosage (CBZ); (b) pH *vs.* catalyst dosage (NFC); (c) pH *vs.* H_2_O_2_ (CBZ); (d) pH *vs.* H_2_O_2_ (NFC); (e) pH *vs.* PS (CBZ); (f) pH *vs.* PS (NFC); (g) catalyst dosage *vs.* H_2_O_2_ (CBZ); (h) catalyst dosage *vs.* H_2_O_2_ (NFC); (i) catalyst dosage *vs.* PS (CBZ); (j) catalyst dosage *vs.* PS (NFC); (k) H_2_O_2_*vs.* PS (CBZ); and (l) H_2_O_2_*vs.* PS (NFC). [EPs concentrations: 2 mg L^−1^ and time: 120 min].

The photo-Fenton-like degradation was strongly influenced by the combined effect of initial pH and catalyst dosage, with optimal performance observed at specific operating conditions. Under UV-A irradiation, the interaction between pH and catalyst dosage confirms that pH is the dominant governing parameter, with catalyst loading exerting a synergistic enhancement. In both processes, the degradation increases sharply as pH approaches neutrality, indicating that radical generation and catalyst surface activity are maximized near pH 7 due to the synergistic Ce^3+/4+^ redox cycling, Pd-assisted electron transfer, and the micro-acidic environment of zeolite Y, which ultimately enables efficient activation of H_2_O_2_ and PS.^70,71^ Increasing the catalyst dosage further improves removal efficiency by providing additional active sites.^72^ However, this effect is more pronounced for NFC removal, which achieves a higher overall efficiency of *Ca.* 95% compared to CBZ, with a removal efficiency of *Ca.* 85%, as illustrated in Fig. 3(a) and (b), respectively. However, under extreme acidic or alkaline conditions, higher catalyst loading cannot compensate for unfavourable reaction conditions, demonstrating that optimal degradation of both pollutants requires a balanced combination of neutral pH and sufficient catalyst availability.

The combined influence of H_2_O_2_ concentration and pH, and of pH and PS, reveals that oxidant activation is strongly pH-dependent for both CBZ and NFC under UV-A irradiation. At extremely low and high pH, the changes in H_2_O_2_ concentration produce only minor improvements in the photo-Fenton-like degradation for both the EPs. Excess H_2_O_2_ does not proportionally enhance performance due to radical scavenging effects [*Cf.*eqn (6)], producing hydroperoxyl radical (HOO˙). The hydroperoxyl radical further quenched the free radicals [*Cf.*eqn (7)], which further confirms the role of optimized H_2_O_2_ concentration in the degradation process, as depicted as shown in Fig. 3(c) and (d), respectively.^73^ Similarly, as depicted in Fig. 3(e) and (f), increasing the PS concentration from 150 mg L^−1^ to 180 mg L^−1^ improves the removal efficiency for both EPs. However, further increasing PS concentration leads to a decline in degradation percentage due to the excess PS and SO_4_˙^−^, which acts as a scavenger, shown in eqn (8) and (9).^74^

6 H_2_O_2_ + ˙OH → H_2_O + ˙OOH

7 ˙OOH + ˙OH → H_2_O + O_2_

8 SO_4_˙^−^ + S_2_O_8_^2−^ → SO_4_^2−^ + S_2_O_8_^˙−^

9 SO_4_^˙−^ + SO_4_^˙−^ → S_2_O_8_^2−^

The interactions between catalyst dosage and H_2_O_2_, and between catalyst dosage and PS concentrations, demonstrate a synergistic enhancement in the removal of CBZ and NFC. As shown in Fig. 3(g–j), increasing catalyst loading, together with H_2_O_2_ and PS concentrations, progressively improves removal efficiency by promoting more effective oxidant activation and radical generation *via* additional active sites. In both cases, NFC consistently achieves higher removal efficiencies at elevated catalyst and oxidant concentrations, indicating the efficient utilization of available active sites. In contrast, CBZ shows a more gradual response and greater sensitivity to catalyst dosage, suggesting that the active sites available play a more limiting role in the degradation process.

Furthermore, the interaction between H_2_O_2_*vs.* PS concentrations reveals a synergistic effect on the degradation efficiency for CBZ and NFC. Fig. 3(k–l) shows that increasing H_2_O_2_ and PS concentrations yields incremental improvements in the degradation of these two pollutants. However, the catalyst performs best for these EPs at balanced oxidant concentrations, as excessive dosing does not significantly enhance overall removal and can even reduce radical utilisation. Both the EPs follow similar interaction patterns, with pH as the primary controlling factor, catalyst dosage as a secondary enhancer, and H_2_O_2_/PS concentrations enhancing optimum behaviour. The perturbation plot from the RSM-CCD analysis further confirms the significant effects of UV-A irradiation on CBZ and NFC, as depicted in Fig. S4(a) and S4(b), respectively. However, NFC demonstrates higher degradation performance and a wider optimal operating range than CBZ, suggesting greater sensitivity to the photo-Fenton-like activation of H_2_O_2_ and PS. The RSM-CCD analysis indicated that the maximum removal for these EPs is achieved under neutral pH with balanced catalyst and H_2_O_2_/PS concentrations, showing degradation efficiencies of 85.82% for CBZ and 96.72% for NFC at neutral pH and 180 mg L^−1^ PS concentration for both the EPs, 0.1 mL L^−1^ and 0.14 mL L^−1^ H_2_O_2_ concentration with 180 mg L^−1^ (CBZ) and 110 (NFC) mg L^−1^ catalyst dosage. Moreover, Fig. S5(a–l) illustrates that the elimination of CBZ and NFC under Vis-light illumination showed similar trends, with slightly reduced efficiencies, achieving removal efficiencies of *Ca.* 79% and 87% for CBZ and NFC, respectively.

### 3.3. Influence of EPs concentration and various co-existing ions

**Fig. 4:**
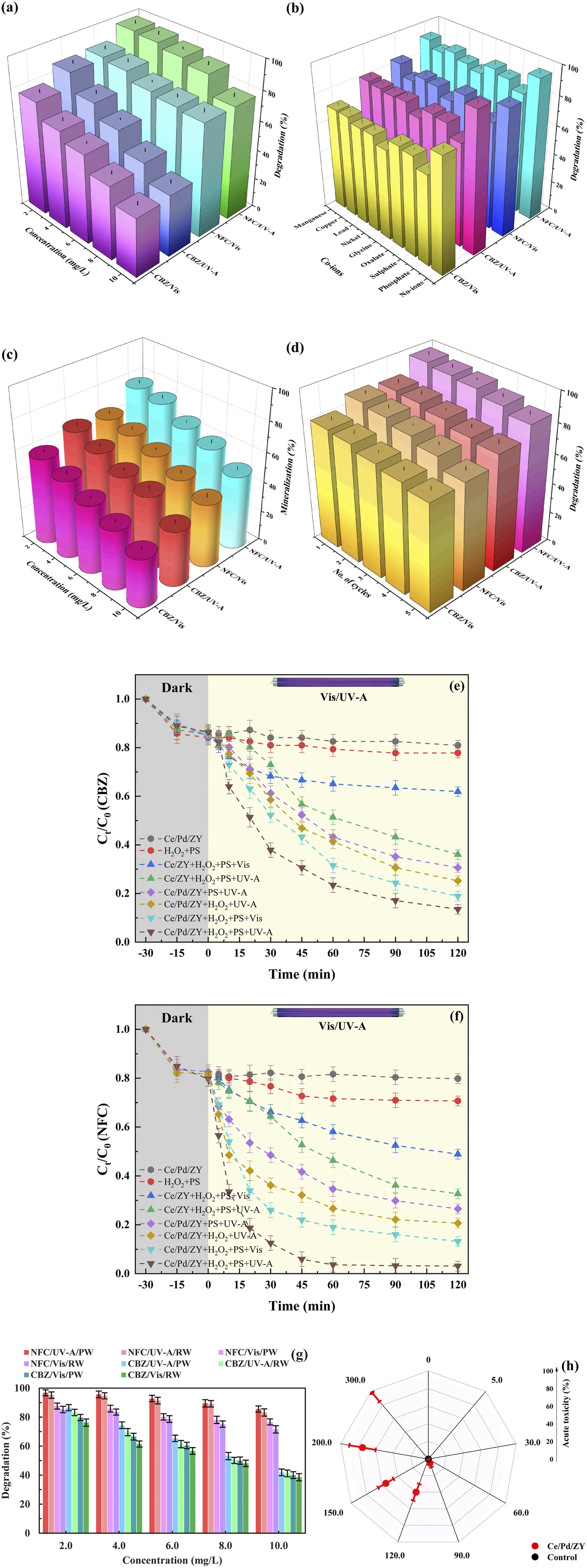
(a) Effect of initial concentration of EPs; (b) influence of selected co-existing ions; (c) mineralization efficiency in the degradation of the EPs; (d) reusability of Ce/Pd/ZY towards the degradation of the selected EPs; (e) photo-Fenton-like degradation of CBZ across various reaction parameters; (f) photo-Fenton-like degradation of NFC across various reaction parameters; (g) evaluation of CBZ and NFC elimination in pure water and real wastewater systems; and (h) acute toxicity test of the Ce/Pd/ZY catalyst [pH: 7; H_2_O_2_ (30%): 0.1 mL L^−1^ (CBZ) and 0.15 mL L^−1^ (NFC); catalyst dosage: 150 mg L^−1^ (CBZ) and 125 mg L^−1^ (NFC); PS: 180 mg L^−1^ (CBZ) and 150 mg L^−1^ (NFC); reaction time: 120 min].

The implication of the EPs concentration on the elimination performance of Ce/Pd/ZY was evaluated over an initial EPs concentration ranging from 2–10 mg L^−1^ utilizing the previously optimized reaction parameters. As shown in Fig. 4(a), increasing the initial EPs concentration reduced elimination efficiency. For CBZ, the removal efficiency decreased from *Ca.* 86% to 41% under UV-A illumination and from 79% to 40% under visible light illumination with an increase in concentration from 2 to 10 mg L^−1^, respectively. A similar trend was obtained for NFC, where degradation efficiency declined from *Ca.* 96% to 85% and from 87% to 76% under UV-A and Vis light irradiations, respectively, over a similar decrease in pharmaceutical concentrations. The decrease in elimination efficacy with increasing pollutant concentrations was attributed to increased competition among pollutant molecules for the finite number of active centres on the Ce/Pd/ZY catalyst surface. As the pollutant concentration increases, a greater number of molecules compete for the same active regions, reducing the availability of active sites for effective catalytic interaction.^75^ Consequently, fewer EPs molecules access the active sites of Ce/Pd/ZY, reducing overall degradation efficiency.

Furthermore, because natural water systems commonly contain various inorganic and organic ions, the potential influence of these ions on the degradation process, was systematically investigated under different light irradiation conditions using the Ce/Pd/ZY catalyst. The tests were conducted in the optimized reaction conditions with the EP : co-ions concentration of 2 : 10 mg L^−1^ to ensure consistency and comparability of results. Fig. 4(b) illustrates the effectiveness and selectivity of the Ce/Pd/ZY catalyst for the elimination of CBZ and NFC in the presence of several co-ions in aqueous solution. Throughout different conditions, only slight variations in degradation efficacy were observed, indicating that the effects of co-existing ions are largely insignificant. However, due to competitive adsorption of co-ions on the catalyst's surface and the potential quenching ability of some co-ions, such as phosphate,^66^ it hinders the elimination of EPs to *Ca.* 71% (CBZ) and *Ca.* 82% (NFC) under UV irradiation. Furthermore, degradation efficiency is even lower under Vis light: 62% (CBZ) and *Ca.* 73% (NFC). In contrast, glycine competes with EPs for reactive species and forms stable complexes with the catalytic metal centres, thereby inhibiting oxidant activation and suppressing degradation performance.^60^ In the presence of glycine, the removal efficiency of CBZ is reduced to *Ca.* 72% (UV-A) and *Ca.* 62% (Vis) while NFC shows removal efficiency of *Ca.* 83% (UV-A) and *Ca.* 74% (Vis).

### 3.4. Mineralization of EPs and reusability of the Ce/Pd/ZY catalyst

The total organic carbon (TOC) analysis was conducted to determine the extent of mineralization of CBZ and NFC in the water treatment process. Fig. 4(c) presents the mineralization efficiencies of the EPs at varied concentrations ranging from 2 to 10 mg L^−1^. The degradation process using the Ce/Pd/ZY catalyst under UV-A irradiation achieved approximately 65% and 76% mineralization of CBZ and NFC, respectively. However, the mineralization efficiency significantly decreased at higher EPs concentrations, and the Vis-light illumination exhibited slightly lower mineralization performance compared to the UV-A irradiated process. The enhanced mineralisation under UV-A is attributed to the higher generation of ˙OH radicals, which promotes the oxidation of intermediate products, resulting in fewer stable by-products.^76^ At higher CBZ concentration (10 mg L^−1^), the efficiency declined to *Ca*. 37% and 49% for CBZ and NFC, respectively. Moreover, under Vis-light illumination, the mineralization of EPs exhibited a similar downward trend, decreasing from 57% to 32% for CBZ and from 64% to 42% for NFC as the EPs concentration was increased from 2 to 10 mg L^−1^. Overall, under both light irradiations, the Ce/Pd/ZY catalyst demonstrates substantial mineralization of CBZ and NFC in the treatment process, highlighting its potential for widespread environmental applications.

The catalyst reusability and long-term operational stability are critical factors in evaluating practical applicability. In particular, a catalyst's ability to maintain reactivity over multiple cycles reflects its reliability for long-term applications. Fig. 4(d) illustrates the reusability performance of Ce/Pd/ZY in the removal of CBZ and NFC under optimized reaction parameters. The results revealed only a marginal decline in degradation efficiency over successive cycles. After five consecutive reuse cycles, CBZ elimination under UV-A irradiation declined from *Ca.* 86% to 77%, while that of NFC declined from *Ca.* 96% to 85%, demonstrating good catalyst durability. Moreover, under Vis-light irradiation, the removal efficiencies of CBZ and NFC declined only slightly, decreasing from *Ca.* 79% to 70% for CBZ and from *Ca.* 87% to 71% for NFC. The excellent structural stability of the Ce/Pd/ZY results in a slight loss of catalytic performance across successive cycles; however, partial catalyst loss during repeated retrieval and reutilization also contributes to a gradual decline in degradation efficiency over multiple catalytic cycles.

### 3.5. Degradation of CBZ and NFC with different experimental parameters

Fig. 4(e) and (f) illustrate the photo-Fenton-like degradation of CBZ and NFC under different reaction conditions using UV-A and Vis light irradiation, respectively. All experiments were conducted under the optimized conditions, including pH, H_2_O_2_/PS concentrations, catalyst dosage, and initial EPs concentration. In both experimental processes, UV-A-driven degradation was more efficient at removing EPs than the Vis-light-driven process. This enhanced degradation of EPs using UV-A light is attributed to the higher photon energy of UV-A irradiation, which more effectively excites electrons and promotes the generation of more reactive species.^77^ Moreover, irradiation of UV-A/Vis-light activates H_2_O_2,_ producing more ˙OH radicals [*Cf.*eqn (10)].^64^

10 H_2_O_2_ + *hv* → 2˙OH

The Ce/Pd/ZY catalyst achieved a high CBZ removal efficiency of *Ca.* 86% in the presence of H_2_O_2_/PS under UV-A irradiation and *Ca.* 79% under Vis-light illumination after 120 min. Similarly, the maximum NFC degradation efficiencies of *Ca.* 96% and *Ca.* 87% were observed under UV-A and Vis light with H_2_O_2_/PS, respectively. The catalytic performance of the Ce/Pd/ZY is primarily governed by the generation and dynamics of electron–hole pairs in an oxygen-rich environment, their recombination rates, and continuous activation of H_2_O_2_ and PS. Overall, the Ce/Pd/ZY catalyst showed superior catalytic activity compared to Ce/ZY, with a CBZ removal efficiency of *Ca.* 63% (UV-A) and *Ca.* 38% (Vis). Moreover, the degradation efficiency of NFC utilizing Ce/ZY showed *Ca.* 67% (UV-A) and *Ca*. 38% (Vis). This is due to the fabrication of Pd NPs into the Ce/ZY catalyst, which resulted in a significant enhancement of EPs degradation due to the synergistic effect arising from the formation of a bimetallic heterojunction.^78^ A similar result was reported where Pd NPs incorporation with FeO_*x*_ shows strong metal–metal support interaction in the activation of H_2_O_2_ for the removal of sulfamethoxazole.^79^ In another study, the presence of Pd^0^ in a Pd-Fe_3_O_4_ system significantly accelerated the activation of peroxymonosulfate due to effective electron transfer in the degradation of trichloroethylene.^80^

### 3.6. Degradation kinetics

The photo-Fenton-like activation of H_2_O_2_ and PS proceeds through a series of elementary reactions, each characterized by its own rate constant. The involvement of multiple parallel and sequential steps renders the overall reaction pathway complex, making the kinetic modelling of the degradation process challenging. Despite this complexity, the apparent rate constant is generally attributed to the interaction of the generated reactive radical species and the EPs.^73^ To evaluate the degradation behaviour, the experimental data were evaluated using both pseudo-first-order (PFO) and pseudo-second-order (PSO) kinetic models, as shown in eqn (S2) and (S3) in the SI document. Comparative fitting indicates that the degradation profiles of the examined EPs are better described by the PFO model than by the PSO model [*Cf.* Fig. S6], suggesting that radical-EP interactions predominantly govern the reaction rate.

### 3.7. Performance of Ce/Pd/ZY on a real water system and toxicity of the catalyst

The degradation efficiency of Ce/Pd/ZY toward CBZ and NFC at different initial spiking concentrations was investigated in real wastewater (RW) in comparison with purified water (PW) under both UV-A and Vis-light irradiation, with the physicochemical properties of RW summarized in Table S12. Following spiking with known concentrations of the EPs, the degradation process was performed utilizing the optimal reaction parameters, and the findings are illustrated in Fig. 4(g). The elimination of CBZ and NFC showed only a slight decrease in efficiency when RW was utilized under UV-A/Vis-light irradiation compared to PW at the same concentration range. The removal efficiencies for CBZ in RW were *Ca.* 83% under UV-A and 76% under Vis-light, whereas NFC exhibited higher removal rates of 95% under UV-A and 85% under Vis-light in the RW at the EPs concentration of 2 mg L^−1^. These findings indicate that activating H_2_O_2_/PS using the Ce/Pd/ZY catalyst is effective and robust for practical applications in treating real wastewater or natural effluent samples. Moreover, the treated samples were also analyzed using ICP-OES before and after the degradation experiments. No detectable Ce or Pd was observed in the solution before treatment, while the treated solution contained Ce and Pd concentrations of 0.01 mg L^−1^ and 0.008 mg L^−1^, respectively, indicating minimal metal leaching and good structural stability of the catalysts.

Moreover, the acute toxicological study of the Ce/Pd/ZY catalyst is depicted in Fig. 4(h), showing an EC_50_ value of 145.6 mg L^−1^ for *Daphnia magna* after a 48 h test period. The 95% confidence interval spans 130.5 to 162.5 mg L^−1^, indicating that the organism is highly insensitive towards the catalyst. This comparatively high EC_50_ value suggests low toxicity and implies minimal adverse impact on aquatic organisms. The toxicity assessment revealed that the Ce/Pd/ZY catalyst exhibited relatively low acute toxicity at lower concentrations, with only 5% mortality observed at a catalyst concentration of 5.0 mg L^−1^. Mortality increased progressively with increasing catalyst dosage, and complete mortality (100%) was recorded only at the highest tested concentration of 300.0 mg L^−1^. These findings suggest that the catalyst poses minimal ecological risk at operating concentrations, although adverse effects become significant at elevated dosages. Previous studies reported the acute toxicity of cerium to be 1.50 mg L^−1^ and 0.014 mg L^−1^ for palladium toward *Daphnia magna*, which is extremely toxic.^81,82^ In contrast, using zeolite Y as a stable support for metal NPs significantly mitigates these concerns. The porous framework of ZY provides a high surface area and promotes the dispersion of NPs, thereby improving durability and suppressing catalyst ion leaching.^58,83^Table 2 further compares the removal of both EPs across different catalysts and systems with high removal efficiency. It is noteworthy that the toxicity of EPs and degradation by-products is occasionally reported; however, the toxicity of the catalyst itself is often not evaluated.

**Table 2:** Comparative table for the removal of CBZ and NFC

Sl. No.	Catalyst	Time (min)	pH	Concentration (mg L^−1^)	Oxidant	EPs	Toxicity tests	Degradation (%)	Ref.
1	Fe_3_O_4_-NP@CNF	60	7	1	H_2_O_2_	CBZ	NA	100	84
2	3DP-Ni/Fe^0^	10	3	10	H_2_O_2_	CBZ	NA	100	85
3	FeS_2_@GO	30	7	10 µg L^−1^	H_2_O_2_	CBZ	NA	100	86
4	Ce/Pd/ZY	120	7	2	H_2_O_2_/PS	CBZ	Experimental & theoretical	86	Present work
5	Fe^2+^	275	3	5 µg L^−1^	H_2_O_2_	NFC	NA	100	87
6	Fe^2+^	60	7	5 µg L^−1^	H_2_O_2_	NFC	NA	100	88
7	FeCo_2_O_4_-C	25	3, 5	10	NA	NFC	Experimental	75	89
8	Ce/Pd/ZY	120	7	2	H_2_O_2_/PS	NFC	Experimental & theoretical	96	Present work

### 3.8. Degradation mechanism and pathway

**Fig. 5:**
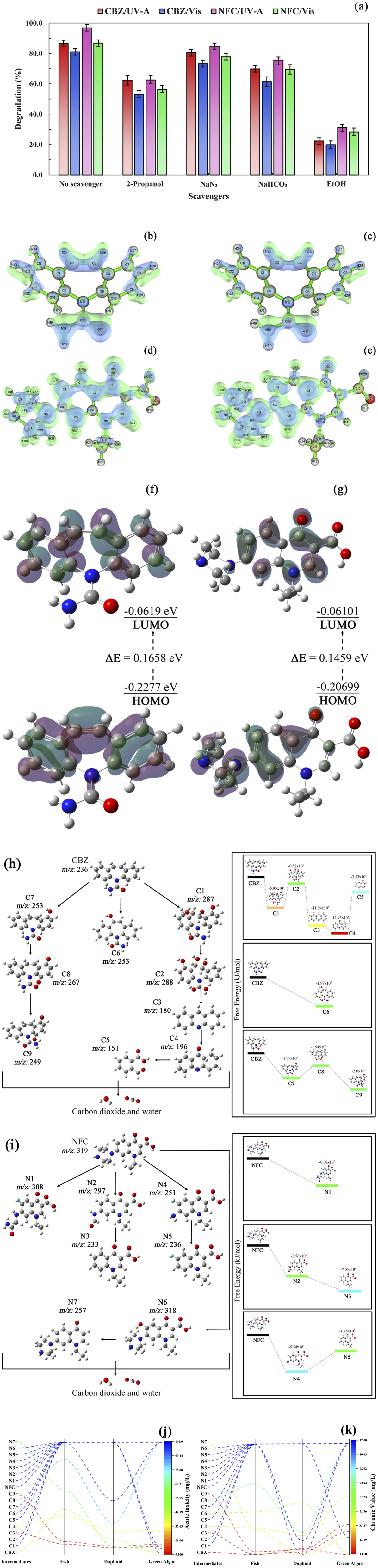
(a) Influence of radical scavengers in the removal of CBZ and NFC under UV-A and Vis-light [pH: 7; H_2_O_2_ (30%): 0.1 mL L^−1^ (CBZ) and 0.15 mL L^−1^ (NFC); catalyst dosage: 150 mg L^−1^ (CBZ) and 125 mg L^−1^ (NFC); PS: 180 mg L^−1^ (CBZ) and 150 mg L^−1^ (NFC); reaction time: 120 min]; (b) *f*^0^ values for CBZ; (c) *f*^−^ values for CBZ; (d) *f*^0^ values for NFC; (e) *f*^−^ values for NFC; (f) HOMO/LUMO energy gap of CBZ; (g) HOMO/LUMO energy gap of NFC; (h) reaction mechanism of CBZ [inset: Δ*G* values]; (i) reaction mechanism of NFC [inset: Δ*G* values]; (j) acute toxicity of CBZ and NFC along with their degradation intermediates [EC_50_/LC_50_]; (k) chronic values of CBZ and NFC along with their degradation intermediates [ChV].

Radical scavenging tests elucidate the dominant reactive species involved in the photo-Fenton-like degradation of CBZ and NFC under UV-A and Vis irradiation. As illustrated in Fig. 5(a), in the absence of scavengers, high degradation efficiencies were achieved (CBZ: *Ca.* 86% UV-A, 81% Vis; NFC: *Ca.* 97% UV-A and 87% Vis), confirming the strong catalytic activity of the Ce/Pd/ZY system. The addition of 2-propanol significantly suppressed CBZ removal efficiency to *Ca.* 62% and 53% under UV-A and Vis-light, respectively, whereas *Ca.* 62% and 56% for NFC, indicating the substantial involvement of ˙OH radicals.^72^ Further, relatively moderate inhibition was observed with NaHCO_3_ for both CBZ and NFC, showing *Ca.* 69% and 75% under UV-A, respectively, while showing *Ca.* 61% and 69% removal efficiencies under Vis-light irradiation, respectively, suggesting the participation of both ˙OH and SO_4_˙^−^ radicals.^90^ However, the effect of NaN_3_ was less pronounced, implying a minor contribution of singlet oxygen (^1^O_2_). Notably, ethanol (EtOH), a non-selective scavenger for both ˙OH and SO_4_˙^−^, shows the highest degradation inhibition towards both the EPs.^91^ With the addition of EtOH, the elimination efficiency of CBZ decreased to *Ca.* 22% and 19% under UV-A and Vis-light, respectively. The elimination of NFC also showed only *Ca.* 31% and 28% under the same light conditions, demonstrating that the radical pathways dominate the degradation process. The stronger suppression by EtOH compared to 2-propanol indicates that SO_4_˙^−^ plays a dominant role, with ˙OH acting as a secondary yet significant oxidizing species. Overall, the results confirm that CBZ and NFC degradation proceed mainly *via* a coupled SO_4_˙^−^/˙OH radical mechanism,^92^ with limited contributions from ^1^O_2_ and photogenerated holes. The proposed mechanistic reaction schemes are shown in eqn (11)–(21).

11 Ce/Pd/ZY + *hv* → e_CB_^−^ + h_VB_^+^

12 Ce^3+^ + S_2_O_8_^2−^ → Ce^4+^ + SO_4_˙^−^ + SO_4_^2−^

13 Pd^0^ + S_2_O_8_^2−^ → Pd^2+^ + SO_4_˙^−^ + SO_4_^2−^

14 e_CB_− + S_2_O_8_^2−^ → SO_4_˙^−^ + SO_4_^2−^

15 Ce^3+^ + H_2_O_2_ → Ce^4+^ + ˙OH + OH^−^

16 Ce^4+^ + H_2_O_2_ → Ce^3+^ + ˙OOH + H^+^

17 Pd^0^ + H_2_O_2_ → Pd^2+^ + ˙OH + OH^−^

18
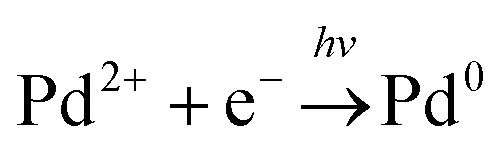

19 SO_4_˙^−^ + H_2_O → ˙OH + HSO_4_^−^

20 SO_4_˙^−^ + OH^−^ → ˙OH + SO_4_^2−^

21 CBZ/NFC + SO_4_˙^−^/˙OH → CO_2_ + H_2_O

To further elucidate the degradation mechanism, density functional theory (DFT) calculations were performed to identify the most reactive sites in CBZ and NFC. Hirshfeld surface analysis was employed to examine the electron density distribution surrounding individual atoms, thereby revealing charge localization within each molecule.^46^ The Fukui indices (FI) were calculated to predict the susceptibility of specific atoms toward radical and electrophilic attack. Atoms exhibiting higher FI of *f*^0^ and *f*^−^ were identified as the most probable reactive centres for radical and electrophilic species, respectively. The calculated FI for CBZ and NFC are presented in Table S13. Nevertheless, we conducted Mayer bond order (MBO) analysis to identify relatively weak bonds more likely to cleave during oxidation (Table S14). We also analysed the distributions of the frontier molecular orbitals (HOMO and LUMO) to assess the molecules' electron-donating/accepting properties. In addition, we further investigated the degradation process using LC-MS analysis and determined the Gibbs free energy changes (Δ*G*). However, the obtained energy differences are not interpreted as Gibbs free energy changes for individual elementary reaction steps; instead, they represent the theoretical relative energies of the identified molecular intermediates.

Table S13 shows that the FI indicators for CBZ are C3, C2, C9, C13, and C7, which represent the most reactive sites toward radical attack [*Cf.*Fig. 5(b)], while similar regions are also favoured for electrophilic attack [*Cf.*Fig. 5(c)], highlighting C3 and C2 as the key reactive centres. In the case of NFC, N12, C9, O22, C5, and C2 exhibit the highest susceptibility to both radical and electrophilic attacks [*Cf.*Fig. 5(d) and (e)], demonstrating that the N12 region plays a dominant role in its reactivity. Moreover, MBO analysis revealed that in CBZ, the C2–H25 and C3–H26 bonds have comparatively low bond orders (0.96243 and 0.94109), indicating weaker bond strength and a higher probability of cleavage during oxidation. Similarly, in NFC, bonds associated with N12–C13 and N12–C15 exhibit relatively lower bond orders, suggesting that these positions are structurally less stable and more vulnerable to degradation. Frontier molecular orbital analysis shows very low HOMO–LUMO energy gaps for both CBZ and NFC (0.1658 and 0.1459 eV, respectively), reflecting their high chemical reactivity, as shown in Fig. 5(f–g). The HOMO electron density is primarily localised over the azepine ring in CBZ and the amine regions in NFC, indicating that these moieties are the most probable sites for oxidation reaction.

The degradation pathways of CBZ and NFC, as identified by LC-MS analysis, are shown in Fig. 5(h) and (i), respectively. The FI and LC-MS analyses collectively showed coherent insight into the degradation mechanism of CBZ in the ˙OH/SO_4_˙^−^ to the system. The FI showed strong localization of electron density over the olefinic C2–C3 bond of the azepine ring and adjacent aromatic carbons. The significant overlap between the *f*^0^ and *f*^−^ reactive regions indicates that these sites are highly prone to both radical and electrophilic attack. Evidently, following the FI analysis, the attack of ˙OH radical on the central 7-membered ring through hydrolysis occurred, producing products C1 and C6. Further reaction led to the formation of C2, in which the –NH_2_ on the azepine rings was replaced by ˙OH. Product C2 further evolved into acridine (C3) and acridone (C4). The weakened electronic structure, as indicated by the FI values at the olefinic carbons, facilitates subsequent C–N and C–C bond cleavage, leading to ring opening into a smaller aromatic fragment and the formation of C5. Continued radical attack promotes further hydroxylation, aromatic ring cleavage, and oxidative fragmentation into lower molecular oxygenated intermediates. Ultimately, repeated oxidation steps mineralize the molecule into CO_2_ and H_2_O. In an alternative pathway, ˙OH radicals initiate the degradation through successive hydroxylation reactions. Initially, ˙OH adds to the benzene ring, forming intermediate C7. This reaction is continued by a second ˙OH addition, yielding the formation of the isomerized product C8. Nevertheless, an N-centred radical is generated, forming C9, due to the intramolecular cyclization reaction, promoted by the reaction between SO_4_˙^−^ and the –NH_2_ functional group.^93^

Moreover, NFC degradation proceeds through oxidation pathways at the most electron-rich sites predicted by the FI. The initial step involves ˙OH addition to the aromatic ring, thereby generating the mono-hydroxylated intermediate (N1). This step increases the polarity of the molecule and destabilizes the aromatic system, making it more susceptible to further oxidation. In a parallel pathway, ˙OH abstracts a hydrogen atom to form intermediate N2. The continued oxidative attack promotes structural rearrangement and partial ring opening, leading to N3. This transformation likely involves the breaking of weakened C–C or C–N bonds, consistent with the predicted low Mayer bond order values. Aromaticity is partially reduced, and oxygen-containing functional groups are introduced. Another degradation pathway begins with successive hydroxylation of the benzene ring, forming N4. Further oxidation yields N5, a dehydroxylated product. Moreover, the increased electron deficiency and ring activation facilitate oxidative ring cleavage, including hydrogen abstraction and β-scission reactions, shortening the carbon framework to produce N7. However, with continued ˙OH attack, these intermediates undergo progressive oxidation, eventually degrading into smaller molecules and finally mineralizing into CO_2_ and H_2_O.

The ecological risks associated with the transformation intermediates formed during the treatment of CBZ and NFC are evaluated using ECOSAR V2.2. The results indicated that most of the intermediates derived from CBZ and AMX exhibit negligible acute toxicity, with predicted LC_50_/EC_50_ values exceeding 100 mg L^−1^ [*Cf.*Fig. 5(j)] and chronic toxicity values exceeding 10 mg L^−1^ [*Cf.*Fig. 5(k)]. Compared with their respective parent compounds, these intermediates exhibit substantially reduced toxic potential. Moreover, as the reaction progressed and treatment duration increased, the intermediate species continued to undergo successive oxidative transformations. Notably, after 120 min of treatment with the Ce/Pd/ZY catalyst, a substantial fraction of the intermediates generated from CBZ and NFC were converted into CO_2_ and H_2_O. These findings indicate the high catalytic efficiency of the Ce/Pd/ZY in promoting effective oxidation and mineralization, thereby ensuring environmentally safe removal of CBZ and NFC from the aqueous environment. Collectively, the results underscore the catalyst's strong potential for sustainable and eco-friendly wastewater treatment applications.

## 4. Conclusion

The current study demonstrates that the bimetallic cluster (Ce/Pd/ZY) effectively activates both H_2_O_2_ and PS under a photo-Fenton-like system for the degradation of CBZ and NFC. The catalyst exhibits high crystallinity, and incorporating Pd increases the surface area and improves its electrochemical and photochemical properties. The synergistic redox interactions between nano-Ce and Pd species enhanced radical generation and promoted efficient pollutant removal under neutral pH conditions by activating H_2_O_2_ and PSO, showing optimal CBZ removal of *Ca.* 86% and *Ca.* 96% compared to NFC. Moreover, real wastewater analysis showed an optimum removal percentage of *Ca.* 83% and 95% for CBZ and NFC, respectively. The degradation kinetics were best described by a PFO model, confirming that the reactions between pharmaceuticals and reactive radicals govern the overall treatment process. The identified intermediates revealed sequential oxidative transformations leading to substantial mineralization after extended treatment time. Moreover, toxicity assessments indicated an EC_50_ of 145.6 mg L^−1^ and reduced ecological risk after treatment, demonstrating the catalyst's low toxicity and effective wastewater detoxification. Overall, the Ce/Pd/ZY catalyst offers a promising, environmentally sustainable approach to eliminating persistent pharmaceutical contaminants from wastewater systems.

## Conflicts of interest

The authors declare that they have no competing interests.

## Supplementary Material

RA-OLF-D6RA05958A-s001

## Data Availability

The data supporting this article are included in the supplementary information (SI) document. Supplementary information is available. See DOI: https://doi.org/10.1039/d6ra05958a.

## References

[cit1] Shearer L., Pap S., Gibb S. W. (2022). J. Environ. Chem. Eng..

[cit2] Wang J., Wang S. (2016). J. Environ. Manage..

[cit3] Werkneh A. A., Gebru S. B., Redae G. H., Tsige A. G. (2022). Heliyon.

[cit4] Xu J., Tran T. N., Lin H., Dai N. (2018). J. Membr. Sci..

[cit5] Mahire S., Tiwana A. S., Khan A., Nalawade P. M., Bandekar G., Trehan N., Mukkannawar U., Kaur S., Pandit V., Kamble P. N. (2023). Arab. J. Geosci..

[cit6] Gierbolini J., Giarratano M., Benbadis S. R. (2016). Expert Opin. Pharmacother..

[cit7] Wang Y., Gao J., Zhou S., Lian M. (2023). Environ. Technol. Innov..

[cit8] Grouli A., Bachra Y., Damiri F., Pandit V., Berrada M. (2023). Biointerface Res. Appl. Chem..

[cit9] Parlapiano I., Prato E., Denti G., Biandolino F. (2024). Water.

[cit10] Somathilake P., Dominic J. A., Achari G., Langford C. H., Tay J.-H. (2018). J. Water Process Eng..

[cit11] Wang C., Hansen H. C. B., Andersen M. L., Strobel B. W., Ma H., Dodge N., Jensen P. E., Lu C., Holm P. E. (2022). J. Hazard. Mater..

[cit12] Adly A., Galal M. M., Matta M. E. (2025). Sci. Rep..

[cit13] Narsude J., Jadhav J., Rena V., Khan A., Chauhan R., Sonawane R., Dhole S., Pandit V., Jadhav A., Awale M., Patidar S. K., Dheravath B., Kamble P. (2025). Curr. Microbiol..

[cit14] Khandarkhaeva M., Batoeva A., Sizykh M., Aseev D., Garkusheva N. (2019). J. Environ. Manage..

[cit15] Southworth B. A., Voelker B. M. (2003). Environ. Sci. Technol..

[cit16] Watwe V. S., Kulkarni S. D., Kulkarni P. S. (2021). ACS Omega.

[cit17] Chen L., Li X., Zhang J., Fang J., Huang Y., Wang P., Ma J. (2015). Environ. Sci. Technol..

[cit18] Chen Y., Miller C. J., Waite T. D. (2021). Environ. Sci. Technol..

[cit19] Lee J., von Gunten U., Kim J.-H. (2020). Environ. Sci. Technol..

[cit20] Yan Y., Wei Z., Duan X., Long M., Spinney R., Dionysiou D. D., Xiao R., Alvarez P. J. J. (2023). Environ. Sci. Technol..

[cit21] Ding J., Shen L., Yan R., Lu S., Zhang Y., Zhang X., Zhang H. (2020). Chemosphere.

[cit22] Cai J., Niu B., Xie Q., Lu N., Huang S., Zhao G., Zhao J. (2022). Environ. Sci. Technol..

[cit23] Ding J., Sun Y.-G., Ma Y.-L. (2021). ACS Omega.

[cit24] Thomas N., Dionysiou D. D., Pillai S. C. (2021). J. Hazard. Mater..

[cit25] Yin Z., Zhu K., Pan H., Hou Z., Chen J., Wang W., Zeng F., Tan M., Lin D., Liu D., Ye X., Xianyu Y., Chen S. (2025). ACS Nano.

[cit26] Mithari J., Pandit V., Gaikwad S. (2026). MRS Commun..

[cit27] Mithari J. A., Pandit V. R. U., Gaikwad S. (2026). J. Indian Chem. Soc..

[cit28] Pandit V. U., Patil P. M., Goncalves G., Gupta M., Hazarika K. P., Late D. J. (2026). Chem. Phys. Rev..

[cit29] Dawange G. P., Pandit V. U. (2026). ChemistrySelect.

[cit30] Dawange G. P., Pandit V. U. (2026). Chem. Eng. J. Adv..

[cit31] Bae E., Samanta P., Yoo J., Jung J. (2016). Ecotoxicol. Environ. Saf..

[cit32] Malhotra N., Ger T.-R., Uapipatanakul B., Huang J.-C., Chen K. H.-C., Hsiao C.-D. (2020). Nanomaterials.

[cit33] Dixit R., Wasiullah, Malaviya D., Pandiyan K., Singh U. B., Sahu A., Shukla R., Singh B. P., Rai J. P., Sharma P. K., Lade H., Paul D. (2015). Sustainability.

[cit34] Jo H.-J., Son J., Cho K., Jung J. (2010). Chemosphere.

[cit35] Ren H., Shen X. (2025). Ecotoxicol. Environ. Saf..

[cit36] Zhang Q., Tan S.-Y., Liu Y.-Y., Cao A., Yang S.-T., Wang H. (2025). NanoImpact.

[cit37] Ramachandran M., Schwabe K. A., Ying S. C. (2021). Environ. Sci. Technol..

[cit38] Barton D. G., Shtein M., Wilson R. D., Soled S. L., Iglesia E. (1999). J. Phys. Chem. B.

[cit39] Serra A., Manno D., Buccolieri A., Carbone G. G., Calcagnile L. (2018). Mater. Res. Express.

[cit40] Garg U. K., Kaur M. P., Garg V. K., Sud D. (2008). Bioresour. Technol..

[cit41] Lv Z.-B., Feng J., Zhao R.-J., Shen J.-J., Yang W.-W. (2024). Chemosphere.

[cit42] Wei Y., Lu G., Xie D., Sun T., Liu Y., Zhang Y., An J., Li M., Guo H. (2022). Chem. Eng. J..

[cit43] Wang Y., Teng C., Begin E., Bussiere M., Bao J. L. (2024). J. Chem. Theory Comput..

[cit44] OECD , OECD Guidelines for the Testing of Chemicals, Section 2, Organisation for Economic Co-operation and Development, 2012, 10.1787/9789264185203-en

[cit45] OECD , OECD Guidelines for the Testing of Chemicals, Section 2, Organisation for Economic Co-operation and Development, 2004, 10.1787/9789264069947-en

[cit46] Lalhruaitluangi M., Ralte L., Tiwari D., Kim D.-J. (2026). Sep. Purif. Technol..

[cit47] Hamilton M. A., Russo R. C., Thurston R. V. (1977). Environ. Sci. Technol..

[cit48] Mohod A. V., Momotko M., Shah N. S., Marchel M., Imran M., Kong L., Boczkaj G. (2023). Water Resour. Ind..

[cit49] Shah N. S., Iqbal J., Sayed M., Rauf S., Albaqami M. D., Khan J. A., Khan Z. U. H., Rehman F., Khan A., Naseem M., Hashmi A. I. (2021). J. Water Process Eng..

[cit50] Aher R., Punde A., Shinde P., Shah S., Doiphode V., Waghmare A., Hase Y., Bade B. R., Jadhav Y., Prasad M., Pathan H. M., Patole S. P., Jadkar S. R. (2022). ACS Omega.

[cit51] Jubu P. R., Yam F. K., Igba V. M., Beh K. P. (2020). J. Solid State Chem..

[cit52] Sánchez-López P., Hernández-Hernández K. A., Fuentes Moyado S., Cadena Nava R. D., Smolentseva E. (2024). ACS Omega.

[cit53] Xiong Y., McLellan J. M., Chen J., Yin Y., Li Z.-Y., Xia Y. (2005). J. Am. Chem. Soc..

[cit54] Li C., Jia X., Su W., Luan X., Lam S.-M., Sin J.-C., Yu X., Liu L., Bao J., Wang R., Chen J., Zhao Z., Zhao Z. (2026). Appl. Catal., B.

[cit55] Veerakumar P., Sangili A., Chen S.-M., Kumar R. S., Arivalagan G., Firdhouse M. J., Hameed K. S., Sivakumar S. (2024). Chem. Eng. J..

[cit56] Jiang S., Li Y., Gao X., Zhang S., Hou H., Gong J. (2026). Appl. Catal., B.

[cit57] Chen A., Ostrom C. (2015). Chem. Rev..

[cit58] Ralte L., Payanthoth N. S., Hmingsangzuali, Jung J., Tiwari D. (2026). Sep. Purif. Technol..

[cit59] Lv J., Kako T., Li Z., Zou Z., Ye J. (2010). J. Phys. Chem. C.

[cit60] Vanlalhmingmawia C., Tiwari D., Kim D.-J. (2023). Environ. Res..

[cit61] Tian J., Zhong J., Xie Z., Cai H., Song L., Lei K., Zhong X., Liu P., Shang S., Ren Q., Huang H., Chen L., Fu M., Ye D. (2026). Appl. Catal., B.

[cit62] Luo Q., Wang H., Wang L., Xiao F.-S. (2022). ACS Mater. Au.

[cit63] van der Wal L. I., Oenema J., Smulders L. C. J., Samplonius N. J., Nandpersad K. R., Zečević J., de Jong K. P. (2021). ACS Catal..

[cit64] Ralte L., Goswami S., Tiwari D., Jung J. (2025). J. Clean. Prod..

[cit65] Mostafalu R., Heydari A., Banaei A., Ghorbani F., Arefi M. (2017). J. Nanostruct. Chem..

[cit66] Long J., Yang G., Li C., Wu Y., Wang L., Han J., Wu X., Zhan J., Huang B., Yin H., Pan X. (2026). Appl. Catal., B.

[cit67] Iqbal J., Shah N. S., Sayed M., Rauf S., Haq Khan Z. U., Niazi N. K., Polychronopoulou K., Howari F., Rehman F. (2022). J. Clean. Prod..

[cit68] Fan G., Li X., Xu C., Jiang W., Zhang Y., Gao D., Bi J., Wang Y. (2018). Nanomaterials.

[cit69] Saikia H., Borah B. J., Yamada Y., Bharali P. (2017). J. Colloid Interface Sci..

[cit70] Seminko V., Maksimchuk P., Grygorova G., Okrushko E., Avrunin O., Semenets V., Malyukin Y. (2021). J. Phys. Chem. C.

[cit71] Ran W., Zhao H., Zhang X., Li S., Sun J.-F., Liu J., Liu R., Jiang G. (2024). Environ. Sci. Technol..

[cit72] Ralte L., Dihingia H., Pathak S., Lalmalsawmdawngliani, Tiwari D. (2024). Groundw. Sustain. Dev..

[cit73] Dihingia H., Tiwari D. (2022). J. Clean. Prod..

[cit74] Honarmandrad Z., Sun X., Wang Z., Naushad M., Boczkaj G. (2023). Water Resour. Ind..

[cit75] Hassani E. M. S., Duarte H., Brás J., Taleb A., Taleb M., Rais Z., Eivazi A., Norgren M., Romano A., Medronho B. (2024). Polymers.

[cit76] Lalmalsawmdawngliani L. R., Lalhriatpuia C., Tiwari D., Kim D.-J. (2025). J. Environ. Chem. Eng..

[cit77] Mahmoud B. H., Ruvolo E., Hexsel C. L., Liu Y., Owen M. R., Kollias N., Lim H. W., Hamzavi I. H. (2010). J. Invest. Dermatol..

[cit78] Fu H., Wang R., Xu Q., Laipan M., Tang C., Zhang W., Ling L. (2021). Appl. Catal., B.

[cit79] Wang C., Zhang X., Li H., Li J., Qian J., Pan B. (2026). Chem. Eng. J..

[cit80] Deng J., Li F., Qi Z., Huang W., Wan Z., Zhang L., Zheng D., Li G., Zhang F. (2024). Appl. Catal., B.

[cit81] Ma Y., Wang J., Peng C., Ding Y., He X., Zhang P., Li N., Lan T., Wang D., Zhang Z., Sun F., Liao H., Zhang Z. (2016). Ecotoxicol. Environ. Saf..

[cit82] Zimmermann S., Wolff C., Sures B. (2017). Environ. Pollut..

[cit83] Olšovská A., Kamlar M., Samanta S., Veselý J., Čejka J., Mazur M. (2026). Catal. Today.

[cit84] Liu K., Yu J. C.-C., Dong H., Wu J. C. S., Hoffmann M. R. (2018). Environ. Sci. Technol..

[cit85] Li D., Fu Y., Hong W., Li S., Qiu M., Yu H., Wang H., Wu J., Yang Q., Yang S., Xu J., Zhang Y., Chen S., Zhong Y., Peng P. (2024). Environ. Sci. Technol..

[cit86] Ahmed Y., Zhong J., Wang Z., Wang L., Yuan Z., Guo J. (2022). Environ. Sci. Technol..

[cit87] Klamerth N., Malato S., Maldonado M. I., Agüera A., Fernández-Alba A. R. (2010). Environ. Sci. Technol..

[cit88] Klamerth N., Malato S., Agüera A., Fernández-Alba A., Mailhot G. (2012). Environ. Sci. Technol..

[cit89] Zhang P.-Y., Wang W., Wang W.-K., Xi J.-R., Wang Z., Shi Y., Bai J.-W., Li W.-W., Xu J., Zhao G. (2025). Environ. Sci. Technol..

[cit90] Vanlalhmingmawia C., Tiwari D. (2023). Environ. Sci. Pollut. Res..

[cit91] Dihingia H., Pathak S., Lalmalsawmdawngliani L., Tiwari D., Kim D.-J. (2023). J. Taiwan Inst. Chem. Eng..

[cit92] Zhang W., Huang Z., Shen H., Gu Z., Xia S., Zhang P., Han W., Li Y., Tang H. (2022). Colloids Surf., A.

[cit93] Zhang Z., Chen H., Wang J., Zhang Y. (2020). Radiat. Phys. Chem..

