# Supplementary material for: Bimetallic coupling in dual oxidant centres for the efficient removal of pharmaceuticals from wastewater: insights into the reaction mechanism
Source: RSC Adv. 2026 Sep 8;16(43):53375–90. doi: 10.1039/d6ra05958a (PMC13551274; doi:10.1039/d6ra05958a)
Supplement: Supplementary file 1 [file RA-016-D6RA05958A-s001.pdf]

Supplementary Information (SI) for RSC Advances.
This journal is © The Royal Society of Chemistry 2026

Bimetallic coupling in dual oxidant centres for the efficient removal of pharmaceuticals from wastewater: Insights into reaction mechanism

Lalruatkima Ralte, Melody Lalhruaitluangi, Vanhmingliana, Barsha Rabha, Diwakar Tiwari*

Department of Chemistry, School of Physical Sciences

Mizoram University, Aizawl-796004 (India)

*Corresponding author: E-Mail: diw_tiwari@yahoo.com (D. Tiwari), Phone: +91-9862323015

ORCID: https://orcid.org/0000-0002-9177-9704

Supplementary information

$$Y = \gamma_0 + \gamma_1 A + \gamma_2 B + \gamma_3 C + \gamma_4 D + \gamma_{12} AB + \gamma_{13} AC + \gamma_{14} AD + \gamma_{23} BC + \gamma_2$$
$$\gamma_{11} A^2 + \gamma_{22} B^2 + \gamma_{33} C^2 + \gamma_{44} D^2 \quad \ldots(S1)$$

Where, Y denotes degradation efficiency, γ_0 is the intercept term, γ_1, γ_2, γ_3, and γ_4 are linear coefficients, γ_{12}, γ_{13}, γ_{14}, γ_{23}, γ_{24}, and γ_{34} are interaction coefficients, while γ_{11}, γ_{22}, γ_{33} and γ_{44} are quadratic coefficients.

$$\ln \frac{C_t}{C_0} = -K_1 t \quad \ldots(S2)$$

$$\frac{1}{C_t} - \frac{1}{C_0} = K_2 t$$

…(S3)

Table S1: Qualitative analysis of different phytochemicals identified in *C. papaya* leaf extract.

Sl. No.	Phytochemicals	Test	Results
**1**	Alkaloids	Mayer's test, Wagner test	Present
**2**	Flavanoids	Ferric chloride test, lead acetate test	Present
**3**	Tannins	Ferric chloride test	Present
**4**	Triterpenes	Salkowski test, Liberman test	Present
**5**	Steroids	Salkowski test	Present
**6**	Saponins	Foam test	Present
**7**	Cardiac glycoside	Keller Killani test	Present
**8**	Diterpenes	Copper acetate test	Absent
**9**	Phlobatannins	HCl test	Absent

Table S2: Charge transfer resistance value estimated for the different GCEs.

Working Electrode	R_{ct} value (Ω)

Bare GCE	13.6×10^3
Ce/ZY GCE	6.792×10^3
Ce/Pd/ZY GCE	0.428×10^3

Table S3: Comparative BET analysis of the ZY, Ce/ZY, and Ce/Pd/ZY catalysts.

Catalyst	BET-surface area m^2/g	Pore volume cm^3/g	Pore size Å
ZY	1.60	0.0040	366.68
Ce/ZY	9.78	0.0164	64.97
Ce/Pd/ZY	36.04	0.0819	45.45

Table S4: Results of the statistical analysis for the degradation of CBZ under UV-A regression model.

Source	Sum of Squares	df	Mean Square	F-value	p-value	
**Model**	20592.4	14	1470.89	106.91	< 0.0001	significant
A-pH	690.56	1	690.56	50.19	< 0.0001	
B-Catalyst dosage	151.96	1	151.96	11.05	0.0046	
C-H_2O_2	23.1	1	23.1	1.68	0.2147	
D-PS	0.2358	1	0.2358	0.0171	0.8976	
AB	23.81	1	23.81	1.73	0.208	
AC	2.02	1	2.02	0.1466	0.7072	
AD	0.354	1	0.354	0.0257	0.8747	
BC	0.0006	1	0.0006	0	0.9947	
BD	4.67	1	4.67	0.3391	0.569	
CD	2.16	1	2.16	0.1571	0.6975	
A^2	7387.94	1	7387.94	537.01	< 0.0001	
B^2	23.41	1	23.41	1.7	0.2118	
C^2	75.57	1	75.57	5.49	0.0333	
D^2	111.98	1	111.98	8.14	0.0121	
**Residual**	206.36	15	13.76			

Lack of Fit	86.14	10	8.61	0.3582	0.9215	not significant
Pure Error	120.23	5	24.05			
**Cor Total**	20798.8	29				

Table S5: Results of the statistical analysis for the degradation of CBZ under Vis-light regression model.

**Source**	**Sum of Squares**	**df**	**Mean Square**	**F-value**	**p-value**	
**Model**	17672	14	1262.28	104.1	< 0.0001	significant
A-pH	552.89	1	552.89	45.6	< 0.0001	
B-Cat Dose	152.02	1	152.02	12.54	0.003	
C-H_2O_2	26.57	1	26.57	2.19	0.1595	
D-PS	5.43	1	5.43	0.4482	0.5134	
AB	20.09	1	20.09	1.66	0.2175	
AC	0.0885	1	0.0885	0.0073	0.933	
AD	7.83	1	7.83	0.6454	0.4343	
BC	1.91	1	1.91	0.1576	0.6969	
BD	18.47	1	18.47	1.52	0.2361	
CD	0.1871	1	0.1871	0.0154	0.9028	
A^2	7065.2	1	7065.2	582.68	< 0.0001	
B^2	37.71	1	37.71	3.11	0.0982	
C^2	102.35	1	102.35	8.44	0.0109	
D^2	51.65	1	51.65	4.26	0.0568	
**Residual**	181.88	15	12.13			
Lack of Fit	81.07	10	8.11	0.4021	0.8968	not significant
Pure Error	100.81	5	20.16			
**Cor Total**	17853.9	29				

Table S6: Results of the statistical analysis for the degradation of NFC under UV-A regression model.

Source	Sum of Squares	df	Mean Square	F-value	p-value	
**Model**	7636.15	14	545.44	218.59	< 0.0001	significant
A-pH	144.22	1	144.22	57.8	< 0.0001	
B-Cat dose	183.3	1	183.3	73.46	< 0.0001	
C-H_2O_2	1.26	1	1.26	0.5045	0.4884	
D-PS	0.1682	1	0.1682	0.0674	0.7987	
AB	0.4658	1	0.4658	0.1867	0.6718	
AC	4.19	1	4.19	1.68	0.2145	
AD	0.1541	1	0.1541	0.0617	0.8071	
BC	0.0116	1	0.0116	0.0046	0.9466	
BD	12.73	1	12.73	5.1	0.0392	
CD	1.72	1	1.72	0.6904	0.4191	
A^2	1773.17	1	1773.17	710.62	< 0.0001	
B^2	39.32	1	39.32	15.76	0.0012	
C^2	41.99	1	41.99	16.83	0.0009	
D^2	0.7122	1	0.7122	0.2854	0.601	
**Residual**	37.43	15	2.5			
Lack of Fit	31.2	10	3.12	2.51	0.1611	not significant
Pure Error	6.22	5	1.24			
**Cor Total**	7673.58	29				

Table S7: Results of the statistical analysis for the degradation of NFC under Vis-light regression model.

Source	Sum of Squares	df	Mean Square	F-value	p-value	
**Model**	7340.29	14	524.31	132.7	< 0.0001	significant
A-pH	159.31	1	159.31	40.32	< 0.0001	
B-Cat dose	121.99	1	121.99	30.88	< 0.0001	
C-H_2O_2	5.04	1	5.04	1.27	0.2767	
D-PS	0.2335	1	0.2335	0.0591	0.8112	
AB	2.1	1	2.1	0.5303	0.4777	

AC	16.02	1	16.02	4.05	0.0624	
AD	0.1106	1	0.1106	0.028	0.8694	
BC	0.3108	1	0.3108	0.0787	0.7829	
BD	25.48	1	25.48	6.45	0.0227	
CD	1.49	1	1.49	0.3782	0.5478	
A^2	1709.63	1	1709.63	432.7	< 0.0001	
B^2	37.86	1	37.86	9.58	0.0074	
C^2	53.47	1	53.47	13.53	0.0022	
D^2	3.68	1	3.68	0.9322	0.3496	
**Residual**	59.27	15	3.95			
Lack of Fit	44.24	10	4.42	1.47	0.3509	not significant
Pure Error	15.03	5	3.01			
**Cor Total**	7399.56	29				

Table S8: Central composite design matrix with variables (A, B, C, and D), actual and predicted degradation percentage of CBZ under UV-A.

Run No.	A: pH	B: Catalyst dosage (mg/L)	C: H_2O_2 (mL/L)	D:PS (mg/L)	Degradation (%)	Predicted Value (%)
1	2	200	0.1	150	40.21	39.51
2	2	200	0.2	150	37.25	37.23
3	2	100	0.1	210	31.3	30.01
4	7	150	0.15	180	83.21	78.82
5	12	200	0.2	210	20.22	21.12
6	12	200	0.1	210	23.54	23.35
7	7	150	0.2	180	79.25	83.09
8	7	200	0.15	180	85.46	84.73
9	7	150	0.15	180	83.31	78.82
10	12	200	0.2	150	21.17	21.40
11	12	100	0.2	150	18.47	16.94
12	12	100	0.1	210	20.84	21.07
13	7	150	0.1	180	85.82	85.35
14	2	200	0.2	210	38.19	36.36
15	2	100	0.2	150	28.76	27.89
16	2	150	0.15	180	27.62	31.61
17	7	150	0.15	180	73.49	78.82
18	12	150	0.15	180	19.85	19.22
19	7	150	0.15	210	70.99	72.13

20	7	100	0.15	180	74.82	78.92
21	12	200	0.1	150	24.52	25.10
22	2	100	0.1	150	30.88	30.19
23	7	150	0.15	180	83.95	78.82
24	12	100	0.1	150	19.89	20.66
25	2	200	0.1	210	35.42	37.17
26	2	100	0.2	210	29.54	29.18
27	7	150	0.15	180	84.11	78.82
28	12	100	0.2	210	19.18	18.82
29	7	150	0.15	180	74.94	78.82
30	7	150	0.15	150	70.13	72.36

Table S9: Central composite design matrix with variables (A, B, C, and D), actual and predicted degradation percentage of CBZ under Vis-light.

Run No.	A: pH	B: Catalyst dosage (mg/L)	C: H_2O_2 (mL/L)	D:PS (mg/L)	Degradation (%)	Predicted Value (%)
1	7	150	0.1	180	79.72	77.63
2	12	150	0.15	180	14.8	12.37
3	7	150	0.15	180	72.66	70.13
4	12	200	0.1	150	20.55	21.61
5	2	200	0.1	150	37.75	36.49
6	7	200	0.15	180	77.66	76.85
7	12	100	0.2	150	15.72	13.40
8	12	200	0.1	210	19.9	19.55
9	2	200	0.2	210	31.4	29.95
10	2	150	0.15	180	19.33	23.45
11	2	100	0.2	210	24.32	23.36
12	2	100	0.1	210	28.42	26.41
13	12	200	0.2	210	18.33	18.17
14	2	100	0.2	150	23.66	23.49
15	7	150	0.15	180	66.87	70.13
16	12	200	0.2	150	18.32	19.81
17	7	150	0.15	210	64.4	65.11
18	7	150	0.15	180	75.55	70.13
19	12	100	0.1	210	17.77	18.82
20	7	150	0.15	150	65.24	66.21
21	7	150	0.15	180	76.44	70.13
22	7	150	0.15	180	66.42	70.13
23	2	200	0.1	210	29.2	31.62
24	7	100	0.15	180	68.54	71.04
25	2	100	0.1	150	26.72	26.98
26	12	100	0.2	210	15.32	16.06

27	2	200	0.2	150	35.33	34.38
28	7	150	0.15	180	67.88	70.13
29	7	150	0.2	180	71.42	75.20
30	12	100	0.1	150	15.66	16.59

Table S10: Central composite design matrix with variables (A, B, C, and D), actual and predicted degradation percentage of NFC under UV-A.

Run No.	A: pH	B: Catalyst dosage (mg/L)	C: H_2O_2 (mL/L)	D:PS (mg/L)	Degradation (%)	Predicted Value (%)
1	7	125	0.15	180	93.73	93.68
2	12	50	0.2	150	62.33	62.14
3	12	50	0.2	210	60.45	59.71
4	2	50	0.2	210	64.42	63.81
5	12	200	0.2	150	53.34	53.58
6	7	125	0.1	180	90.25	89.92
7	7	125	0.15	180	93.55	93.68
8	12	200	0.2	210	54.82	54.71
9	7	125	0.15	180	94.48	93.68
10	2	125	0.15	180	72.44	70.35
11	2	200	0.1	150	59.48	59.71
12	7	125	0.15	210	92.25	49.11
13	12	200	0.1	210	56.23	54.93
14	2	200	0.1	210	60.92	61.76
15	12	200	0.1	150	51.22	52.49
16	7	200	0.15	180	88.61	86.59
17	12	50	0.1	210	59.5	59.82
18	2	50	0.1	210	66.72	65.96
19	7	125	0.15	180	91.47	93.68
20	2	50	0.2	150	65.85	66.64
21	7	125	0.15	180	92.82	93.68
22	12	50	0.1	150	61.96	60.94
23	7	125	0.2	180	89.62	89.39
24	2	200	0.2	150	58.42	58.76
25	2	50	0.1	150	66.72	67.48
26	7	125	0.15	180	94.33	93.68
27	12	125	0.15	180	63.16	64.69
28	7	50	0.15	180	91.52	92.97
29	2	200	0.2	210	58.99	59.50
30	7	125	0.15	150	96.72	94.30

Table S11: Central composite design matrix with variables (A, B, C, and D), actual and predicted degradation percentage of NFC under Vis-light.

Run No.	A: pH	B: Catalyst dosage (mg/L)	C: H_2O_2 (mL/L)	D:PS (mg/L)	Degradation (%)	Predicted Value (%)
1	12	125	0.15	180	54.92	54.36
2	2	50	0.2	150	54.72	55.59
3	12	50	0.1	150	50.7	50.70
4	7	125	0.15	180	82.87	83.03
5	7	50	0.15	180	80.66	81.81
6	7	125	0.2	180	78.08	77.95
7	12	200	0.2	210	45.89	45.32
8	7	125	0.15	180	81.25	83.03
9	2	50	0.1	150	56.97	57.76
10	7	125	0.15	180	79.42	88.03
11	7	125	0.1	180	80.75	79.01
12	7	125	0.15	210	82.72	84.10
13	2	125	0.15	180	61.62	60.31
14	7	125	0.15	180	84.61	83.03
15	12	50	0.2	210	49.12	49.00
16	12	50	0.1	210	47.96	48.39
17	7	125	0.15	150	87.58	84.33
18	2	50	0.2	210	53.05	52.39
19	12	50	0.2	150	53.6	52.53
20	2	200	0.1	150	50.66	51.03
21	2	200	0.2	210	49.9	50.15
22	2	50	0.1	210	57.17	55.78
23	7	125	0.15	180	82.53	83.03
24	2	200	0.1	210	52.82	54.10
25	12	200	0.2	150	42.16	43.80
26	12	200	0.1	150	41.65	42.52
27	7	125	0.15	180	81.88	83.03
28	12	200	0.1	210	45.88	45.26
29	7	200	0.15	180	79.61	76.60
30	2	200	0.2	150	48.52	48.30

52 Table S12: Physical and chemical properties of the real wastewater.

Source	GPS	Physical and Chemical Properties (mg/L)	pH
SIPMIU	Latitude: 23.730563<br>Longitude: 92.734943	Nickel: 0.544 mg/L<br>Lead: 0.305 mg/L<br>Calcium: 151 mg/L<br>Magnesium: 23.23 mg/L<br>Aluminium: 0.056 mg/L<br>Manganese: 0.055 mg/L<br>Iron: 0.032 mg/L	7.2

Phosphate: 2.122 mg/L
Sulphate: 28.928 mg/L
Nitrate: 134.42 mg/L
Fluorine: 2.88 mg/L
Total Carbon: 485.7 mg/L
Inorganic Carbon: 87.86 mg/L
Total Organic Carbon: 397.9 mg/L
Oxidation Reduction Potential: 165.3 mV
Conductivity: 818 µS/cm
Resistivity: 0.0015 MΩcm
Total Dissolved Solids: 410 ppm
Salinity: 0.52 PSU

Table S13: Fukui Index calculated for the CBZ and NFC.

	CBZ			NFC	
**Atom**	f^0	f^-	**Atom**	f^0	f^-
**C1**	0.0399	0.0379	**C1**	0.0488	0.0342
**C2**	0.0963	0.0999	**C2**	0.0558	0.0536
**C3**	0.0965	0.0978	**C3**	0.0456	0.0666
**C4**	0.0401	0.0417	**C4**	0.0351	0.0379
**C5**	0.0339	0.0303	**C5**	0.0634	0.0816
**C6**	0.035	0.0326	**C6**	0.022	0.025
**C7**	0.0475	0.0506	**C7**	0.0499	0.0228
**C8**	0.0326	0.0313	**C8**	0.0318	0.0179
**C9**	0.065	0.0677	**C9**	0.0722	0.0169
**C10**	0.0324	0.0353	**C10**	0.0182	0.0088
**C11**	0.0473	0.0485	**N11**	0.0262	0.0084
**C12**	0.0332	0.0297	**N12**	0.0769	0.1384
**C13**	0.0645	0.0637	**C13**	0.0165	0.0272
**C14**	0.0307	0.0325	**C14**	0.0125	0.022
**C15**	0.0085	0.0116	**C15**	0.0161	0.0272
**C16**	0.0111	0.0118	**C16**	0.0125	0.022
**O17**	0.0193	0.0201	**N17**	0.0135	0.0251
**N18**	0.0108	0.0092	**C18**	0.0081	0.004
**H19**	0.0083	0.0061	**C19**	0.0053	0.0029
**H20**	0.0078	0.007	**O20**	0.025	0.0137
**H21**	0.0171	0.0172	**O21**	0.0134	0.0071
**H22**	0.0254	0.0238	**O22**	0.0671	0.0426
**H23**	0.017	0.016	**H23**	0.0053	0.003
**H24**	0.0217	0.0209	**H24**	0.005	0.0035
**H25**	0.0384	0.0377	**H25**	0.0053	0.0025
**H26**	0.0385	0.0373	**H26**	0.0102	0.0051
**H27**	0.0217	0.0217	**H27**	0.0095	0.0051
**H28**	0.0168	0.0168	**H28**	0.01	0.0179

**H29**	0.0252	0.0251	**H29**	0.0101	0.0172
**H30**	0.0172	0.0181	**H30**	0.0115	0.0195
			**H31**	0.0127	0.0204
			**H32**	0.0182	0.0305
			**H33**	0.0113	0.0195
			**H34**	0.0108	0.0175
			**H35**	0.0189	0.0319
			**H36**	0.0136	0.0212
			**H37**	0.023	0.0227
			**H38**	0.0259	0.0195
			**H39**	0.0217	0.025
			**H40**	0.011	0.0033
			**H41**	0.0301	0.0086

Table S14: Mayer bond order calculated for the CBZ and NFC.

**CBZ**			**NFC**		
**Bond**		**Bond order**	**Bond**		**Bond order**
**C1**	**C2**	0.96644	**C1**	**C2**	1.8279
**C1**	**C3**	-0.19083	**C1**	**C3**	0.81951
**C1**	**C4**	0.33188	**C1**	**C4**	0.195
**C1**	**C5**	1.54688	**C1**	**C5**	0.11567
**C1**	**C6**	0.14024	**C1**	**C7**	-0.22941
**C1**	**C7**	-0.08019	**C1**	**C8**	-0.14391
**C1**	**C11**	1.12173	**C1**	**O22**	0.05304
**C1**	**C12**	-0.21202	**C1**	**H38**	0.92383
**C1**	**C13**	0.22428	**C2**	**C3**	-0.30165
**C1**	**C14**	0.21875	**C2**	**C4**	1.56397
**C1**	**N15**	-0.08771	**C2**	**C7**	-0.08372
**C1**	**N18**	0.05218	**C2**	**N12**	-0.1701
**C1**	**H24**	-0.19234	**C2**	**C15**	-0.06273
**C2**	**C3**	1.86055	**C2**	**O22**	0.0951
**C2**	**C4**	-0.34295	**C2**	**H37**	0.93623
**C2**	**C5**	0.15348	**C3**	**C5**	0.15205
**C2**	**C6**	0.44543	**C3**	**C6**	1.42269
**C2**	**C9**	0.05977	**C3**	**C7**	0.89119
**C2**	**C12**	-0.16886	**C3**	**C8**	0.15825
**C2**	**H24**	0.08546	**C3**	**C9**	0.23834
**C2**	**H25**	0.96243	**C3**	**N11**	-0.16665
**C3**	**C4**	0.99527	**C3**	**O22**	-0.27613
**C3**	**C5**	0.13783	**C4**	**C5**	1.75533
**C3**	**C6**	0.07752	**C4**	**C6**	0.3927
**C3**	**C7**	-0.05746	**C4**	**C7**	-0.16124
**C3**	**C8**	-0.10199	**C4**	**C8**	-0.07449
**C3**	**C9**	-0.06778	**C4**	**N12**	1.0243
**C3**	**N15**	-0.0706	**C4**	**C15**	0.09687

**C3**	**H26**	0.94109	**C4**	**H34**	-0.05049
**C3**	**H27**	0.09299	**C5**	**C6**	1.41301
**C4**	**C5**	0.11214	**C5**	**C7**	-0.05227
**C4**	**C6**	1.63421	**C5**	**C9**	-0.06526
**C4**	**C7**	1.06506	**C5**	**N11**	-0.12542
**C4**	**C8**	-0.27984	**C5**	**N12**	-0.12238
**C4**	**C9**	0.17391	**C5**	**C13**	0.0671
**C4**	**C10**	0.32733	**C5**	**C18**	0.06071
**C4**	**C11**	-0.08285	**C5**	**H39**	0.92387
**C4**	**N15**	-0.24476	**C6**	**C7**	0.111
**C4**	**C16**	0.062	**C6**	**C8**	0.14207
**C4**	**O17**	-0.10865	**C6**	**C9**	0.11313
**C4**	**H27**	-0.19828	**C6**	**N11**	0.74918
**C4**	**H30**	0.0731	**C6**	**C19**	-0.06915
**C5**	**C6**	0.83936	**C7**	**C8**	0.97989
**C5**	**C8**	-0.08487	**C7**	**C9**	0.40277
**C5**	**C10**	-0.14528	**C7**	**C10**	-0.18446
**C5**	**C11**	0.34763	**C7**	**O21**	0.07189
**C5**	**C12**	0.24065	**C7**	**O22**	1.92494
**C5**	**C13**	-0.1513	**C8**	**C9**	1.85535
**C5**	**C14**	1.17982	**C8**	**C10**	0.66527
**C5**	**N15**	0.83613	**C8**	**C19**	-0.05573
**C5**	**N18**	0.1507	**C8**	**O21**	-0.19109
**C6**	**C7**	0.51454	**C8**	**O22**	-0.21937
**C6**	**C8**	0.22721	**C8**	**H40**	0.06772
**C6**	**C9**	-0.13069	**C8**	**H41**	0.10573
**C6**	**C10**	1.10996	**C9**	**C10**	0.24665
**C6**	**C11**	-0.06745	**C9**	**N11**	1.10434
**C6**	**C12**	-0.06054	**C9**	**O21**	0.06776
**C6**	**C14**	-0.08086	**C9**	**O22**	-0.09865
**C6**	**N15**	0.66444	**C9**	**H41**	0.82717
**C6**	**O17**	0.45034	**C10**	**O20**	1.79527
**C6**	**N18**	-0.08217	**C10**	**O21**	1.12312
**C6**	**H30**	-0.06132	**N11**	**C18**	0.81554
**C7**	**C8**	1.40847	**N11**	**C19**	-0.06288
**C7**	**H27**	1.02832	**N11**	**H26**	-0.06717
**C8**	**C9**	1.37734	**N12**	**C13**	0.8748
**C8**	**H28**	0.97919	**N12**	**C15**	0.88706
**C9**	**C10**	1.53001	**C13**	**C14**	0.68778
**C9**	**H29**	0.97026	**C13**	**C16**	0.06504
**C10**	**N15**	-0.15924	**C13**	**N17**	-0.09115
**C10**	**C16**	-0.06256	**C13**	**H29**	-0.08403
**C10**	**O17**	-0.06936	**C13**	**H31**	0.97575
**C10**	**H30**	0.92498	**C13**	**H32**	1.04151
**C11**	**C12**	1.38973	**C14**	**C15**	0.05033
**C11**	**H24**	1.02275	**C14**	**C16**	-0.16344
**C12**	**C13**	1.35317	**C14**	**N17**	1.02905
**C12**	**H23**	0.98654	**C14**	**H29**	1.02206

**C13**	**C14**	1.55401	**C14**	**H30**	1.0161
**C13**	**H22**	0.96899	**C14**	**H32**	-0.07473
**C14**	**N15**	-0.16478	**C15**	**C16**	0.72125
**C14**	**C16**	-0.06656	**C15**	**H34**	-0.05862
**C14**	**H21**	0.91192	**C15**	**H35**	1.0508
**N15**	**C16**	0.94789	**C15**	**H36**	0.95094
**N15**	**O17**	-0.15094	**C16**	**N17**	1.01536
**N15**	**N18**	-0.13151	**C16**	**H33**	1.0094
**C16**	**O17**	1.68572	**C16**	**H34**	0.99674
**C16**	**N18**	1.24595	**C16**	**H35**	-0.08541
**N18**	**H19**	0.88301	**N17**	**H28**	0.91604
**N18**	**H20**	0.8702	**C18**	**C19**	0.78809
			**C18**	**H26**	1.00634
			**C18**	**H27**	0.97143
			**C19**	**H23**	0.98897
			**C19**	**H24**	0.95995
			**C19**	**H25**	1.00224
			**C19**	**H26**	-0.05064
			**O21**	**H40**	0.84924

Table S15: Quantitative peak fitting parameters of Ce/Pd/ZY.

**Elements**	**Binding Energy (eV)**	**FWHM (eV)**	**Area (%)**
C 1s	284-292	2.75	22.38
O 1s	529-535	3.00	64.02
Ce 3d	880-920	5.75	5.71
Pd 3d	335-345	5.75	7.89

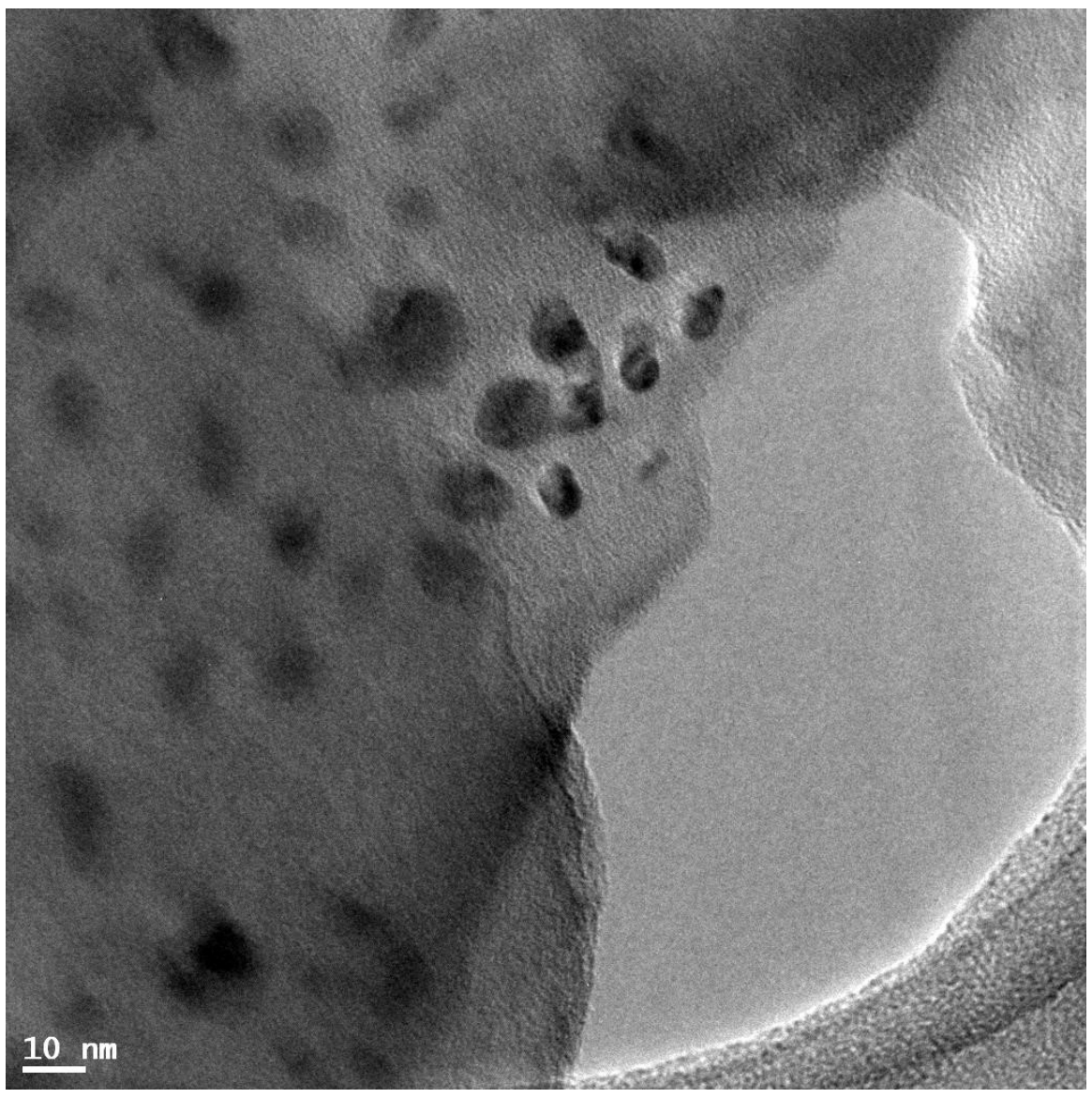

Fig. S1: HR-TEM image of the Ce/Pd/ZY catalyst.

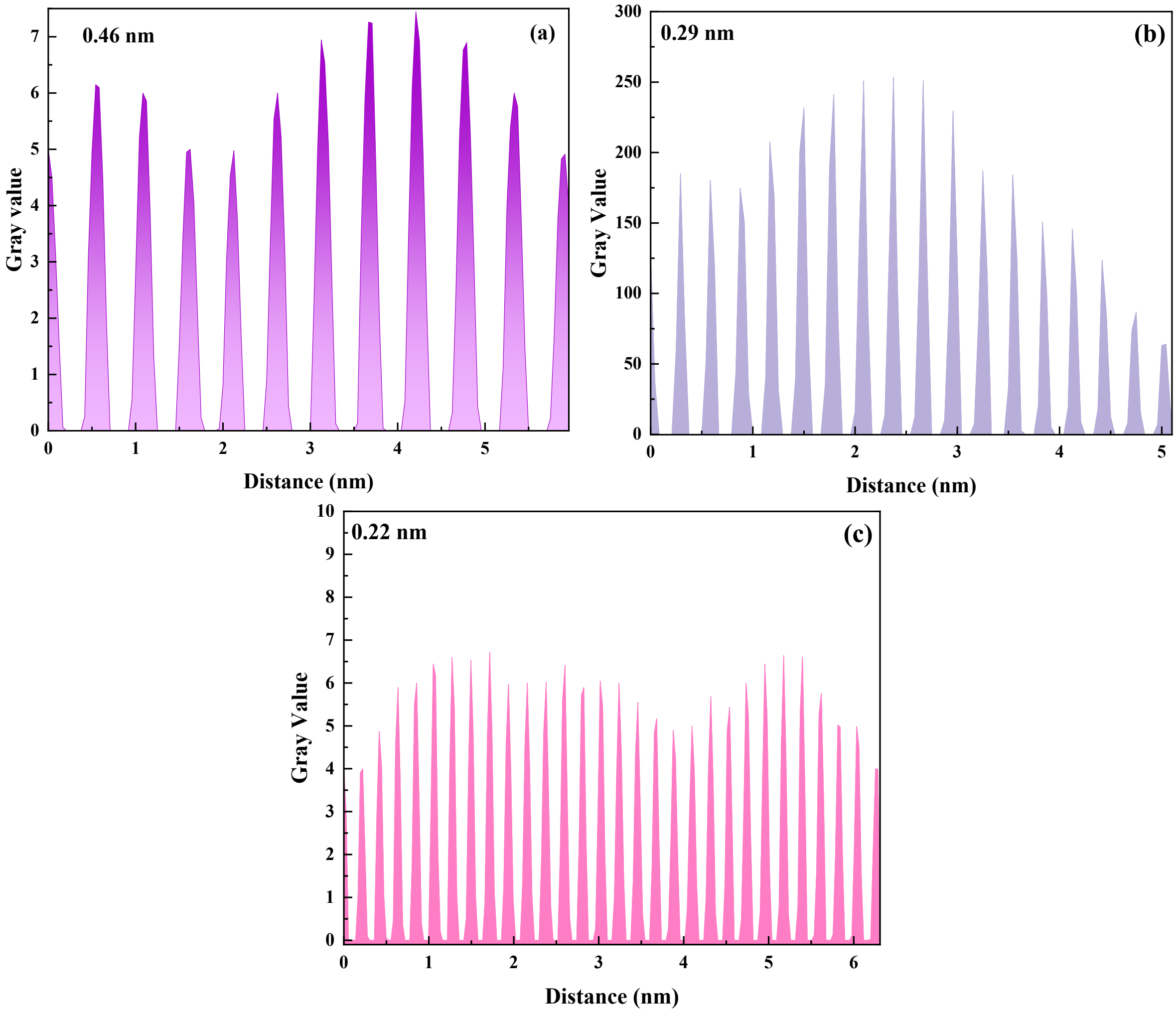

Fig. S2: D-spacing values of: (a) ZY; (b) Ce/ZY; and (c) Ce/Pd/ZY catalysts.

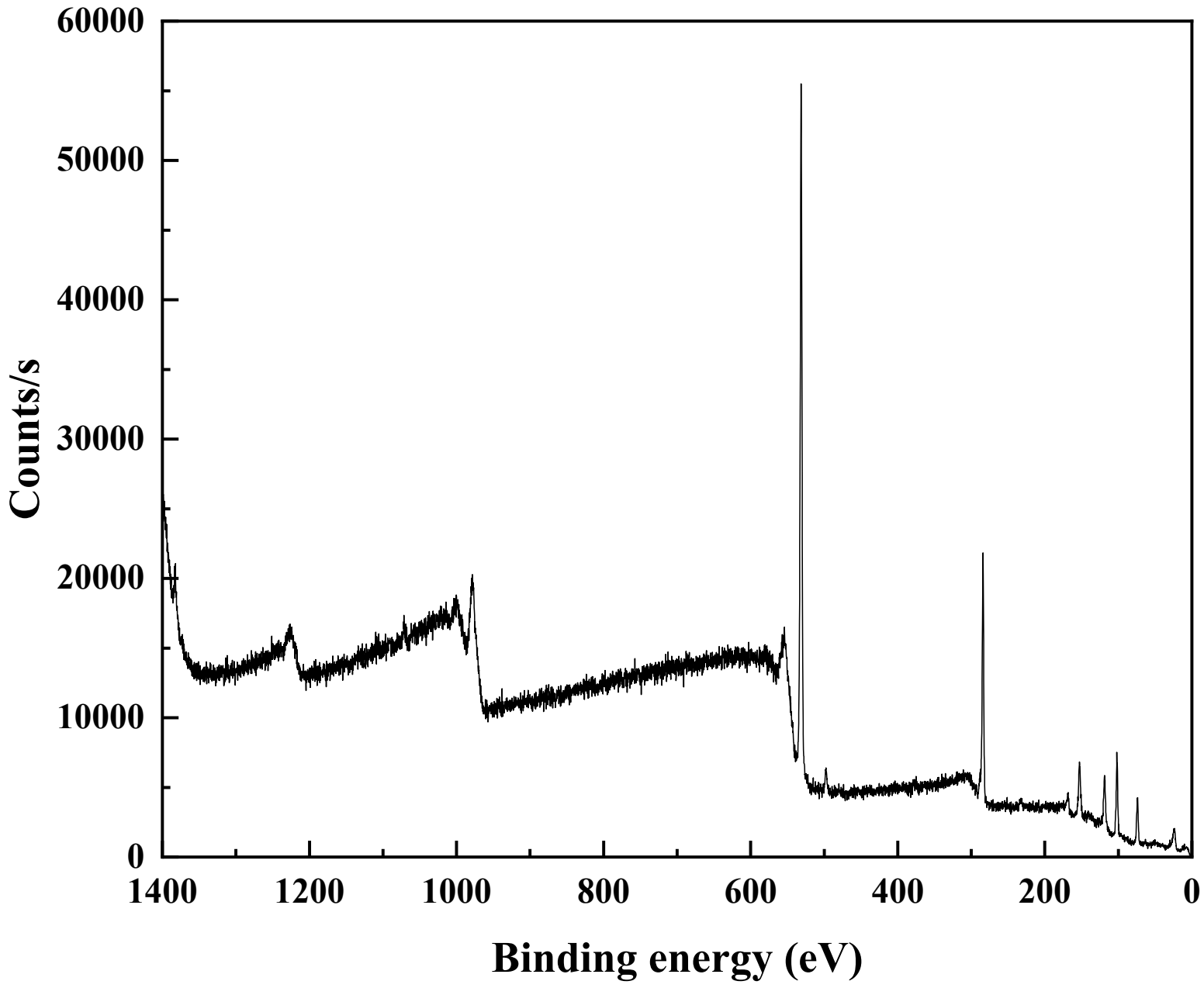

Fig. S3: XPS survey spectrum of the Ce/Pd/ZY catalyst.

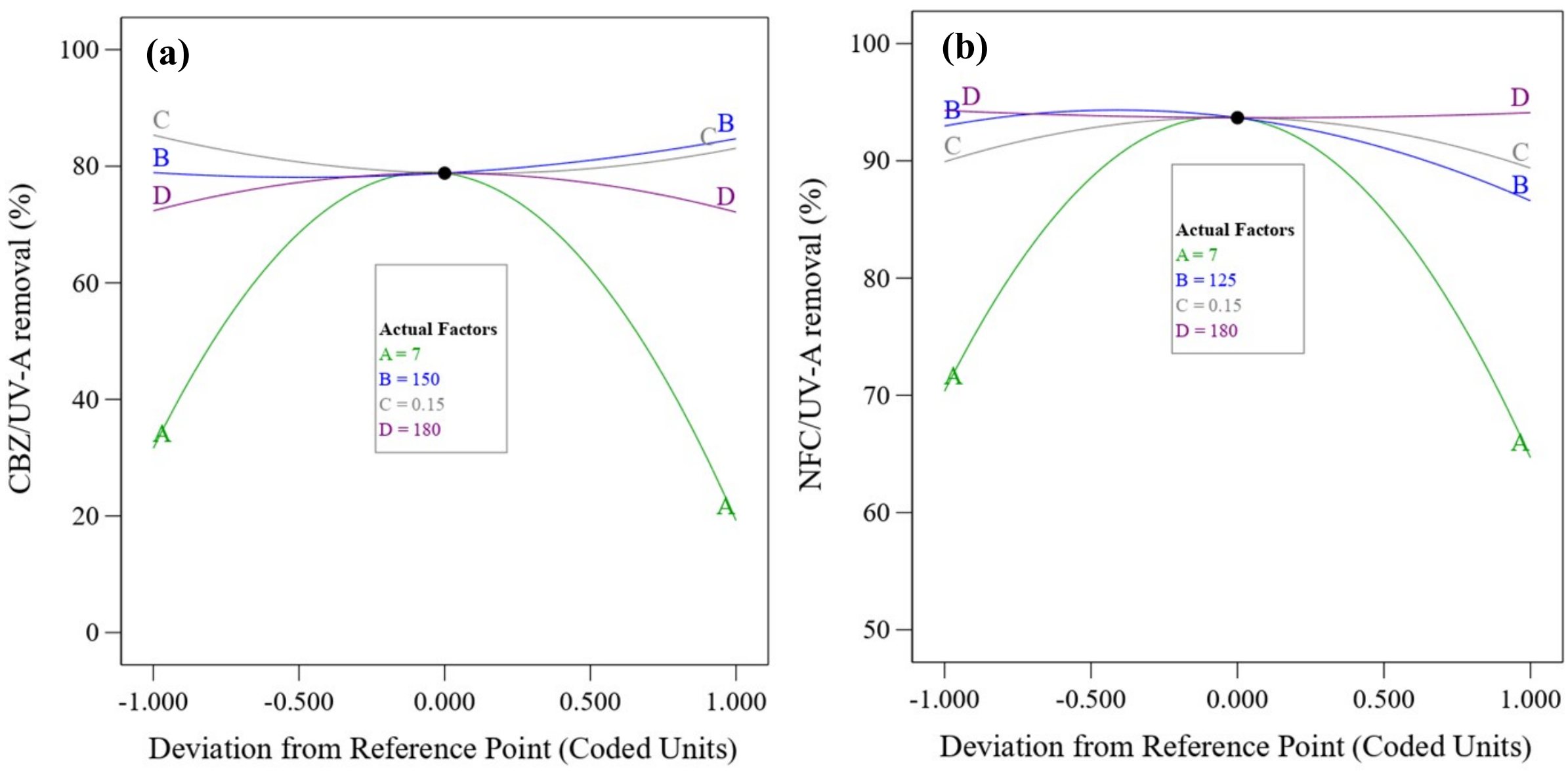

Fig. S4: Variables with a substantial impact on the degradation process on perturbation plot of: (a) CBZ; and (b) NFC under UV-A irradiation.

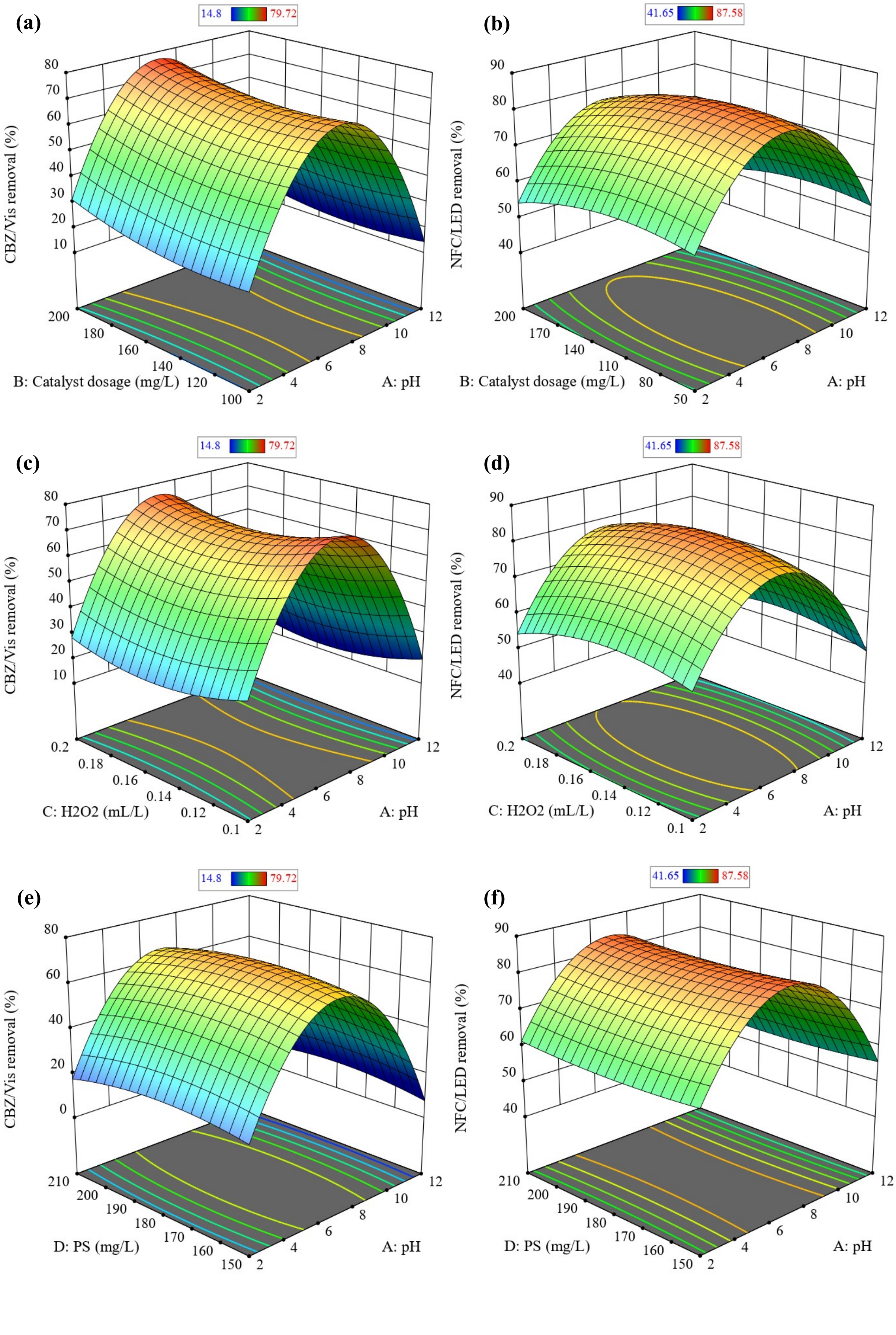

(a)
14.8
79.72
CBZ/Vis removal (%)
B: Catalyst dosage (mg/L)
A: pH
(b)
41.65
87.58
NFC/LED removal (%)
B: Catalyst dosage (mg/L)
A: pH
(c)
14.8
79.72
CBZ/Vis removal (%)
C: H2O2 (mL/L)
A: pH
(d)
41.65
87.58
NFC/LED removal (%)
C: H2O2 (mL/L)
A: pH
(e)
14.8
79.72
CBZ/Vis removal (%)
D: PS (mg/L)
A: pH
(f)
41.65
87.58
NFC/LED removal (%)
D: PS (mg/L)
A: pH

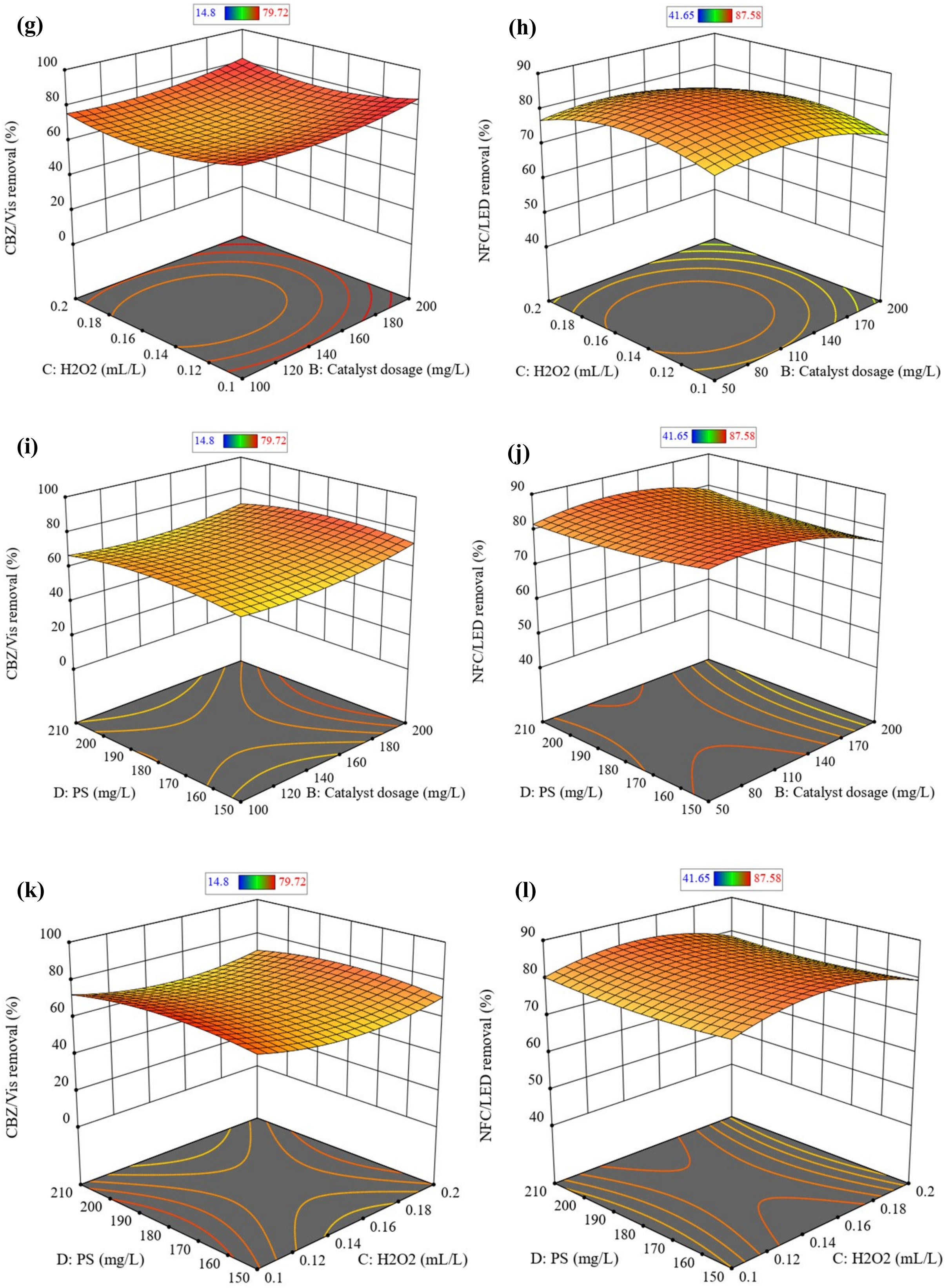

Fig. S5: Degradation efficiency of CBZ and NFC under Vis-light in a 3D surface plot between two factors: (a) pH *vs* catalyst dosage (CBZ); (b) pH *vs* catalyst dosage (NFC); (c) pH *vs* H_2O_2 (CBZ); (d) pH *vs* H_2O_2 (NFC); (e) pH *vs* PS (CBZ); (f) pH *vs* PS (NFC); (g) catalyst dosage

vs H_2O_2 (CBZ); (h) catalyst dosage vs H_2O_2 (NFC); (i) catalyst dosage vs PS (CBZ); (j) catalyst dosage vs PS (NFC); (k) H_2O_2 vs PS (CBZ); and (l) H_2O_2 vs PS (NFC). [EPs concentrations: 2 mg/L and reaction time: 120 mins].

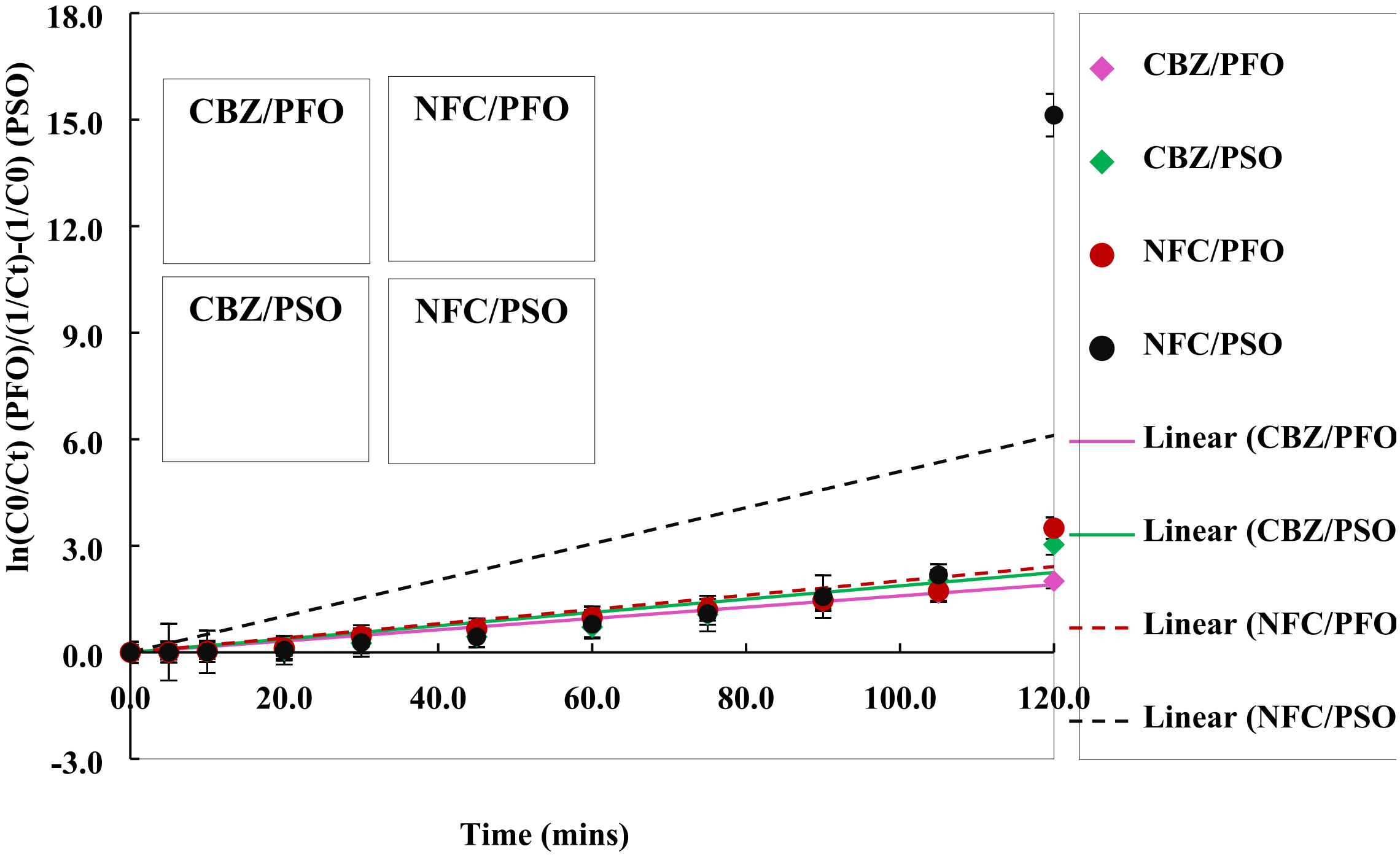

Fig. S6: The PFO and PSO kinetic models for CBZ and NFC.
